# Identification of amino acid metabolism-related gene Leucyl-tRNA synthetase 1 (LARS1) as a potential prognostic and therapeutic target in hepatocellular carcinoma

**DOI:** 10.3389/fonc.2025.1675018

**Published:** 2025-09-16

**Authors:** Qiyu Shi, Yirong Chi, Ziyi Peng, Chao Li, Jingwen Zhao, Jie Zhang

**Affiliations:** ^1^ Department of Gastroenterology and Hepatology, Tianjin Medical University General Hospital, Tianjin, China; ^2^ Department of Gastroenterology and Hepatology, Tianjin Medical University General Hospital, Tianjin Medical University, Tianjin Institute of Digestive Diseases, Tianjin Key Laboratory of Digestive Diseases, Tianjin, China; ^3^ Department of General Surgery, Cangzhou People’s Hospital, Hebei, China

**Keywords:** LARS1, HCC, amino acid metabolism, prognosis model, autophagy

## Abstract

**Introduction:**

Amino acid metabolism plays a critical role in tumorigenesis in hepatocellular carcinoma (HCC). Thus, we explore the amino acid metabolic profile in HCC to construct effective prognosis model and identify novel potential therapeutic target for HCC.

**Methods:**

The transcriptomic data and clinical information of HCC patients were directly obtained from The Cancer Genome Atlas (TCGA). Then we classified them into two subtypes based on selected amino acid metabolism-related genes (SARGs) and explored the differences between them. Besides, risk models were constructed based on SARGs through LASSO regression, and we further validated and evaluated the predictive effect of the model. Subsequently, we validated the key gene of LARS1 in the model. We analyzed the discrepancy of LARS1 in tumor and adjacent non-tumor tissues in both TCGA and the Gene Expression Omnibus (GEO) database and the results were verified in HCC patients undergoing hepatectomy from our hospital via PCR and Immunohistochemistry (IHC). Finally, we explored the biological function of LARS1 *in vitro*.

**Results:**

We classified HCC patients into Cluster A and B subtypes based on 81 SARGs. And patients in Cluster B exhibited significantly poorer prognosis, higher tumor malignancy levels, higher TIDE scores and T cell exhaustion or dysfunction. Then 15 genes were included to construct the risk model. The risk score was positively associated with poor prognosis. We further extracted LARS1 as the key gene of the model and found that high LARS1 tended to have poorer prognosis with higher expression in tumor tissues than in adjacent non-tumor ones in both TCGA and GEO. PCR and IHC were conducted for verification. Suppression of LARS1 markedly inhibited the growth of HCC cells. Additionally, LARS1 knockdown significantly impeded cellular migration and invasion *in vitro*, with increased autophagy flux.

**Conclusion:**

We have successfully developed a prognostic model based on 15 genes associated with amino acid metabolism. We also verified that knockdown of LARS1 significantly inhibited the proliferation, invasion and migration of HCC *in vitro*, with increased autophagy flux, indicating that LARS1 could be a potential therapeutic target for HCC.

## Introduction

Hepatocellular carcinoma (HCC) is reported to be one of the most prevalent cancers worldwide and ranks as the third leading cause of cancer-related death. Globally, there is an estimated of 840,000 new cases and over 780,000 deaths each year (1). HCC is a highly heterogeneous disease with various etiological factors, including chronic hepatitis virus infection, excessive alcohol consumption, autoimmune hepatitis and metabolic disorders. Due to its insidious onset and lack of symptoms in the early stage, HCC is usually diagnosed at an advanced stage when treatment options are limited, albeit with high morbidity and mortality (2). Current therapeutic strategies of HCC include liver transplantation, surgical resection, radiotherapy, chemotherapy, targeted therapies and immunotherapy (3). However, the survival time of HCC patients is only extended by a few months and the overall prognosis remains unsatisfactory. Consequently, there is an urgent need to investigate the intrinsic molecular features of HCC, identifying novel and effective therapeutic targets.

Metabolic reprogramming, a hallmark of cancer, is a key process by which tumor cells support their rapid proliferation and evade immune surveillance (4), including enhanced glycolysis, increased fatty acid synthesis, amino acid metabolism and nucleotide biosynthesis (5). Amino acids are not only the building blocks of proteins but also serve as intermediates in various biosynthetic pathways to produce energy. Multiple studies have highlighted the critical role of amino acid metabolism reprogramming in tumors (6). For instance, glutamine was proved to serve as a vital nutrient for many cancers, supplying both carbon and nitrogen to support various cellular functions, including HCC, which lead to liver cancer cells being resistant to sorafenib (7). In addition, urea cycle dysregulation characterized by alteration from arginine synthesis toward pyrimidine biosynthesis triggers a General Control Nonderepressible 2 (GCN2) kinase-mediated stress response under arginine deprivation, leading to inhibition of HCC cell proliferation (8). And methionine metabolites of S-adenosylmethionine (SAM) and 5-methylthioadenosin (MTA) may promote T cell exhaustion in HCC (9). In human HCC cells and animal models, suppression of branched-chain amino acid (BCAA) catabolic enzyme expression strongly correlated with tumor aggressiveness, and was an independent predictor of clinical outcome (10). Thus, there is an urgent need to explore the amino acid metabolic profile in HCC to improve prognosis and treatment sensitivity for HCC patients.

Leucyl-tRNA synthetase 1 (LARS1) gene encodes a cystosolic leucine-tRNA synthetase, a member of aminoacyl-tRNA synthetases (ARSs). The ARS family are evolutionary conserved enzymes and catalyze the ligation of tRNAs with their cognate amino acids for translation in protein synthesis, playing a pivotal roles in translation of RNA into proteins (11). Expect for aminoacylation of tRNA, ARSs also exhibit important function in various physiological and pathological process, such as angiogenesis, cysteine polysulfidation, immune response and tumorigenesis (12). The non-classical function of LARS1 was reported in many studies. LARS1 was found to bind to Rag GTPase by sensing intracellular leucine concentration, and function as GTPase-activating protein (GAP) for Rag GTPase leading to the mechanistic target of rapamycin complex 1 (mTORC1) activation. The oncogenic effect of LARS1 by the activation of mTORC1 was demonstrated in lung cancer cells (13, 14). Besides, as a leucine sensor, LARS1 has been revealed to regulate leucine metabolism in a glucose-dependent manner. Under conditions where both glucose and leucine are abundant, LARS1 catalyzes the binding of leucine to tRNA, thereby participating in the translation process. However, under glucose-deprived conditions, UNC51-like autophagy-activating kinase (ULK1) phosphorylates LARS1, reducing its ability to bind leucine to save energy (15). As to HCC, it is demonstrated that higher LARS1 expression level was observed in tumor tissues with poor prognosis and correlated with AFP, histologic grade, pathologic stage and so on (16). However, the effect of LARS1 associated with amino acid metabolism on HCC is still elusive.

As a result, this study focuses on exploring the amino acid metabolic profile and elucidating the oncogenic roles of LARS1 in amino acid metabolism of HCC. The ultimate goal is to identify novel prognostic biomarkers and uncover potential therapeutic targets to improve patient prognosis and treatment responsiveness in HCC.

## Materials and methods

### Data acquisition and procession

The transcriptomic data and clinical information for TCGA-LIHC were directly obtained from the GDC portal (https://gdc.cancer.gov/). The raw count data were normalized to TPM (transcripts per million) to represent gene expression, and low-quality genes were filtered out to ensure the quality of the analysis. 366 amino acid metabolism-related genes (ARGs) were extracted from the MSigDB database (https://www.gsea-msigdb.org/) (**Supplementary Table S1**). We further identified 81 selected amino acid metabolism-related genes (SARGs), which have significant impact on prognosis and exhibit discrepancy expression levels between tumor and adjacent non-tumor tissues in HCC (**Supplementary Table S2**).

### Consensus clustering

Initially, we performed consensus clustering analysis using ConsensusClusterPlus R package to cluster HCC patients into distinct molecular subtypes based on the expression of SARGs. We calculated the cumulative distribution function (CDF) for each cluster number K using the consensus matrix to determine the optimal number of clusters. Heterogeneity between different molecular groups was described by principal components analysis (PCA). And Kaplan-Meier (K-M) curves were used to assess the survival between different subtypes based on survival and survminer R package.

### Differential enrichment analysis of SARG subtypes

We identified differential expression genes (DEGs) between the molecular subtypes by edgeR package, with the criteria of | log2FC|≥1 and FDR<0.05. To explore the differences in biological functions between different cluster groups, Gene Ontology (GO) and Kyoto Encyclopedia of Genes and Genomes (KEGG) pathway enrichment analyses were conducted using the clusterProfler R package. To further explore the possible critical pathways in tumor progression across subtypes, gene set variation analysis (GSVA) of Hallmark pathways was performed. Besides,the difference in tumor mutation burden (TMB) level was visualized via maftools R package between different SARG subtypes.

### Tumor microenvironment and drug sensitivity analysis of SARG subtypes

The single gene set enrichment analysis (ssGSEA) was employed to investigate immune cell infiltration within the tumor microenvironment (TME) in HCC patients of SARGs-related subtypes (17). To evaluate the response to immunotherapy in different molecular subtype, the TIDE database (http://tide.dfci.harvard.edu/) was utilized. Then Chi-square tests were conducted to confirm the differences of immunotherapy response between distinct molecular subtypes. Furthermore, we examined T-cell state score (TCSS) through TCellSI package to assess eight distinct T cell states including Quiescence, Regulating, Proliferation, Helper, Cytotoxicity, Progenitor exhaustion, Terminal exhaustion, and Senescence (18). In addition, we obtained drug sensitivity-related data from the Genomics of Drug Sensitivity in Cancer (GDSC2, https://www.cancerrxgene.org/), including 969 cell lines and 297 drugs, and calculated the half maximal inhibitory concentration (IC50) values of the patients between different SARG subgroups using oncoPredict R package.

### Construction of the prognostic model based on SARGs

We established the prognostic risk model for HCC patients based on SARGs by LASSO regression. The risk score formula were established as follows: Risk score=∑(expi*coefi), where expi represents gene expressions and coefi represents regression coefficients. Then patients were categorized into high- and low-risk group according to the median risk score. And we drew heatmap to visualize the expression levels of model genes in high- and low-risk group.

### Validation and evaluation of prognostic risk model

Receiver-operator characteristic (ROC) curves were conducted to verify the accuracy of the risk model. Overall survival (OS) were evaluated by K-M survival analysis based on ‘survminer’ and ‘survival’ R package to evaluate the ability to discriminate between patients of different risk levels. And Wilcoxon tests were conducted to evaluate the differences of risk score between alive and dead HCC cohorts. To further confirm the model’s independent prognostic ability, both univariate and multivariate Cox regression were performed after evaluation of proportional hazards assumption by Schoenfeld residuals test. A nomogram combining the model with clinicopathological features was used to calculate the predicted survival time of HCC patients, and the accuracy was measured via the calibration plot. We also drew Sankey diagram to evalute the relationship among molecular subtypes, risk groups and survival status. Then DEGs between high-risk and low-risk groups were identified based on edgeR package using |log2FC|>1 and FDR<0.05 as criteria. And GO and KEGG were utilized to explore the biological functions of DEGs between high- and low-risk group. Additionally, drug sensitivity was assessed based on GDSC database using oncoPredict R package between the two risk groups. To further extracted the key gene of the risk model, we evaluated the expression levels between tumor and adjacent non-tumor tissues and gene-related prognosis of all model genes.

### Functional analysis related to LARS1 levels

The discrepancy of LARS1 in tumor and adjacent non-tumor tissues were verified in datasets of GSE112790, GSE39791, GSE45267, GSE69715, GSE76427 from the Gene Expression Omnibus (GEO). We classified HCC patients into high- and low-LARS1 groups based on the median LARS1 level. The ‘survminer’ and ‘survival’ R package were used to evaluate prognosis between different LARS1 subgroups. GO, KEGG and GSEA enrichment analyses were also conducted based on DEGs between patients with high- and low-LARS1 levels.

### Single-cell RNA sequencing analysis

Single-cell RNA sequencing data of HCC were obtained in dataset of GSE149614 from GEO. Data processing and downstream analysis were conducted using the Seurat (v4.1.1) R package. The filtering threshold was set as follows:

Excluding cells with fewer than 500 or more than 30,000 of total Unique Molecular Identifier (UMI) counts.

Excluding cells fewer than 200 or more than 8,000 detected genes.

Excluding cells with >10% mitochondrial gene expression.

Gene expression data were normalized according to the Log Normalization algorithm. Batch effect correction among different HCC samples was performed using Harmony. PCA was performed to determine significant and influential dimensions. And the top 10 principal components were clustered via the FindNeighbors and FindClusters functions with a resolution of 0.3. Uniform Manifold Approximation and Projection (UMAP) was used for visualization.

Cell type annotation was performed based on the expression of well-established marker genes. Specifically, hepatoma cells were identified by AFP, EPCAM, ALB and GPC3; T cells by CD3D, CD3E and TRAC; NK cells by NKG7, GNLY and KLRD1; neutrophils by S100A8, S100A9 and FCGR3B; macrophages by CD68, CD14 and CD163; fibroblasts by COL1A1, FAP and THY1; B cells by CD19, CD79A and MS4A1; and endothelial cells by PECAM1, VWF and CDH5. These marker genes were curated from CellMarker (19). Differential expression analysis was performed using the FindAllMarkers function in Seurat to identify cluster-specific marker genes, with the following parameters: logfc.threshold= 0.25, min.pct= 0.1, and only.pos= TRUE. Genes with |log2FC|>1 and FDR< 0.05 were considered statistically significant.

### RNA isolation and quantitative real-time PCR

Total RNA was isolated from fresh cancer and adjacent non-tumor tissues of 3 HCC patients who underwent hepatectomy in Cangzhou People’s Hospital, using an RNA extraction kit (Sparkjade, China). The Ethics Committee of Cangzhou People’s Hospital approved the acquisition of tissue samples and clinical data (Approval No: AF/SC-08/02.0). cDNA was synthesized from the isolated RNA by reverse transcription according to the instructions of the qRT-PCR kit (Takara, Japan). The amplification reactions were carried out using designed primers according to the manufacturer’s protocol (Vazyme). The primer sequences were as follows: LARS1 (5’TTTGCTGTAGGGTACCAGCG3’, 5’CGACGCCAGTCTACCTTCAA3’),β actin (5’TCATCACCATTGGCAATGAG3’, 5’CACTGTGTTGGCGTACAGGT3’).

### Immunohistochemistry

The paraffin-embedded tissue sections were obtained from HCC patients undergoing hepatectomy in Cangzhou People’s Hospital from 2023-2024. The specimens were subjected to high-temperature (70°C) baking for 2 hours, followed by dewaxing with xylene and graded ethanol. Antigen retrieval was performed using citrate buffer solution, and endogenous peroxidase was blocked with 3% hydrogen peroxide. The tissue sections were incubated with a 1:200 dilution of anti-LARS1 antibody (Proteintech, 21146-1-AP, China) to detect the expression of LARS1. The assessment of LARS1 expression levels was performed by two pathologists in a blinded manner. Tumor samples were divided into high and low LARS1 expression groups based on the median expression levels.

### Cell culture and lentiviral transfection

Hep3B, MHCC97H, PLC/PRF/5 (PLC), SK-hep-1, and Hep G2 liver cancer cell lines were obtained from the American Type Culture Collection (ATCC). These cell lines were cultured in Dulbecco’s Modified Eagle Medium (DMEM) supplemented with 10% fetal bovine serum (FBS) and 1% streptomycin-penicillin, and maintained in an incubator at 37°C with 5% CO_2_. The SK-hep-1 cell line was used to construct LARS1 knockdown cell lines. The cells were seeded in 6-well plates at a density of 1 × 10^5^ cells per well. After adherence, shRNA lentivirus was mixed with HiTransG A (GENE, China) and added to the cells. The cells were then treated with 2 μg/mL puromycin (Invitrogen, USA) for at least one week to establish stable cell lines. The shRNA sequences were as follows: sh-LARS1: 5’-CCTCACTTTGACCCAAGCTAT-3’ (sense) and 5’-ATAGCTTGGGTCAAAGTGAGG-3’ (antisense).

### Western blot

The cells were lysed using 1× SDS lysis buffer (62.5 mM Tris-HCl, pH 6.8, 2% SDS, 10% glycerol) supplemented with 1 mM sodium fluoride, 1 mM sodium vanadate, and a 1×protease and phosphatase inhibitor cocktail (Roche, Switzerland) at 4°C for 30 minutes. The collected proteins were denatured in a 95°C water bath for 10 minutes, followed by centrifugation at 13,000×g for 15 minutes at 4°C. Equal amounts of the protein supernatant were loaded onto an SDS-polyacrylamide gel for separation via SDS-PAGE. The proteins were then transferred to a PVDF membrane (Merck, Germany) and blocked with 3% bovine serum albumin (BSA). The membrane was subsequently incubated with primary and secondary antibodies. The following antibodies were used: anti-LARS1 (1:1000) from Proteintech, anti-ATG5 (1:1000) from Cell Signaling Technology, anti-Beclin1 (1:1000) from Cell Signaling Technology, anti-SQSTM1/p62 (1:1000) from Cell Signaling Technology and anti-β-actin (1:1000) from Abcam.

### CCK8 assay

Cells were cultured in a 96-well plate at a density of 1000 cells per well, with 100 μl of cell culture medium and 10 ul CCK8 reagent (Biosharp, China) added to each well. The cells were incubated at 37°C for 3 hours. The absorbance was measured at 450 nm using a microplate reader (Biotek, USA). Six parallel wells were set up for each group, and the average value was calculated. The cell proliferation curve was drawn by continuous detection for3-4 days.

### Edu assay

Cells were seeded in a 6-well plate at a density of 1×10^5^ cells/well and cultured to 70% confluence, then washed with PBS. According to the manufacturer’s instructions (Beyotime, China), fresh DMEM containing 10 μM EdU was added, and the cells were incubated at 37°C with 5% CO_2_ for 2 hours. After incubation, the medium was removed by washing the cells with PBS. The cells were then fixed and stained using the BeyoClick™ EdU-594 Cell Proliferation Kit (Beyotime, China) according to the protocol. A fluorescence microscope was used to capture random fields, and the number of EdU-positive cells was counted.

### Invasion and migration assays

Cell migration and invasion assays were evaluated using Transwell chambers (Neuro Probe, USA). Cells were seeded in a 24-well plate and cultured to 70% confluence. The cell suspension was prepared at a concentration of 1×10^6^ cells/mL in serum-free DMEM and added to the upper chambers of the Transwell system. For the invasion assay, 10% FBS-containing DMEM was added to the lower chambers as the chemoattractant. The upper chambers were precoated with Matrigel (Invitrogen, USA). For the migration assay, the lower chambers were filled with DMEM containing only 10% FBS. The chambers were incubated at 37°C in a 5% CO_2_ incubator.Distances were measured at time points of 36 and 24 hours for invasion and migration assays respectively.

### Statistical analysis

All experimental measurements were were detected in triplicate. Statistical analyses were performed using GraphPad Prism 9.5.0 and R (R-4.3.0). For two group comparison, the Student’s t-test was conducted for normally distributed continuous data and Wilcoxon rank-sum test for others. For multiple group comparisons, one-way analysis of variance (ANOVA) was applied for normally distributed continuous data and the Kruskal-Wallis test for others. Chi-square test was used to compare the differences for categorical variables. LSD *post-hoc* test was used for further pairwise comparisons between group means. The Spearman rank correlation and Pearson correlation coefficients were used to assess the relationship between two variables. Statistical significance was defined as *P*< 0.05, *P*< 0.01, *P*< 0.001, and *P*< 0.0001, with *P*≥ 0.05 indicating no significant difference (ns).

## Results

### Identification of amino acid metabolism-related genes and subtypes

**Figure 1:**
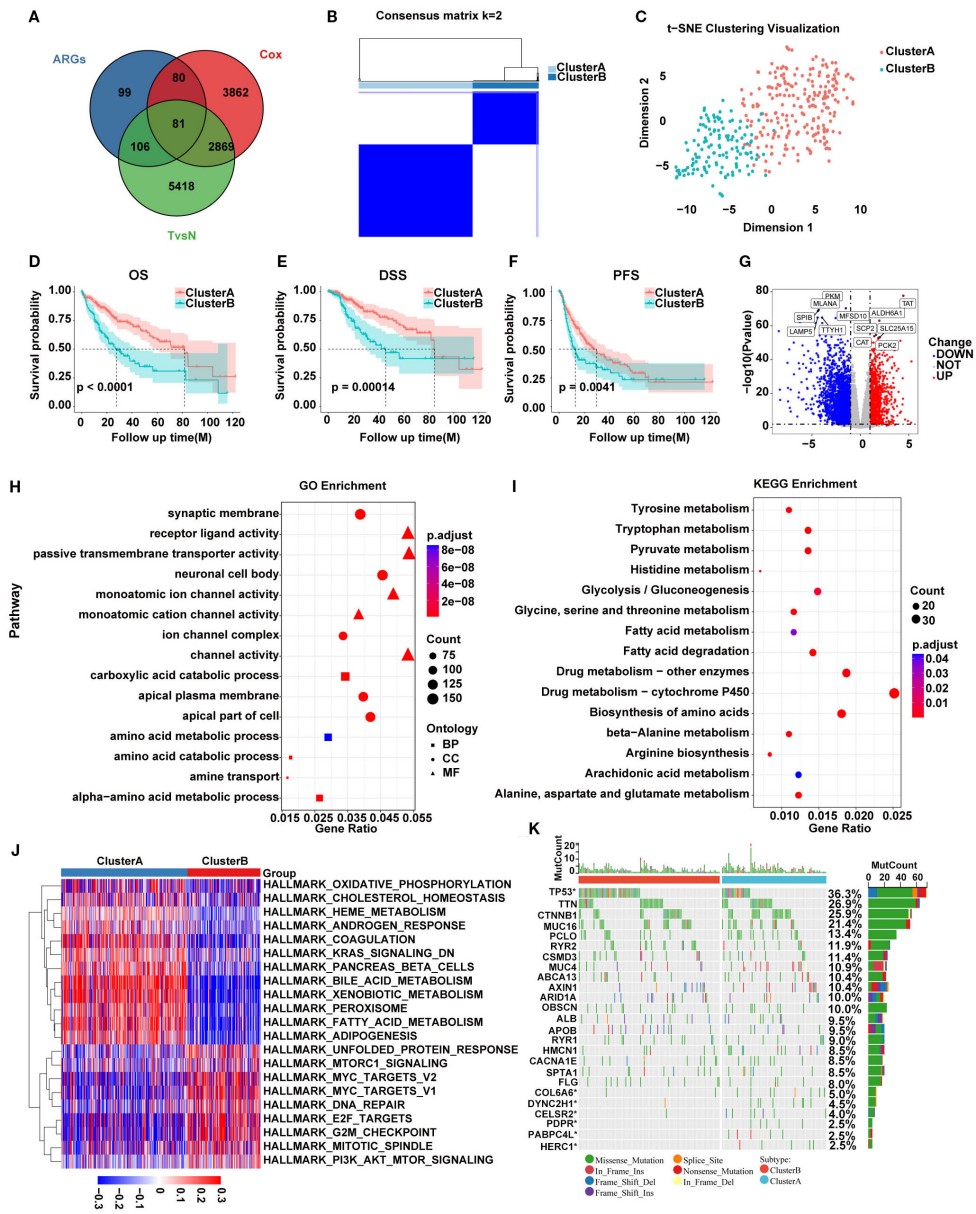
**(A)** Venn diagram showing 81 SARGs after intersection. **(B)** Consensus matrix plot defining the two subtypes. **(C)** PCA showing the differences between the two SARG subtypes. **(D–F)** K-M curves for the two SARG subtypes. **(G)** Volcano plot identifying DEGs between the two SARG subtypes. **(H, I)** GO and KEGG enrichment of DEGs between two SARG subtypes. **(J)** Heatmap showing pathways associated with malignancy progression based on GSVA analysis. **(K)** The distribution of somatic mutations in the two SARG subtypes.

A total of 366 ARGs were obtained from MsigDB website and 81 SARGs were selected after intersecting ARGs, DEGs between HCC tumor and adjacent non-tumor tissues and prognostic genes by Cox regression in 369 HCC patients from TCGA. (**Supplementary Tables S1, S2**; **Figure 1A**). Then TCGA cohort were classified into two HCC subtypes, Cluster A (n=234) and B (n=135) based on the expression of 81 SARGs. (**Figure 1B**). The CDF curve shows a smooth rise, indicating that the clustering results are stable and the correlation between groups is the largest whole be the smallest between groups (**Supplementary Figure S1**). The PCA plot verified the clustering accuracy (**Figure 1C**).

### Functional analysis of SARGs-related molecular subtypes

Survival analysis revealed that Cluster A exhibited significantly better OS, disease-specific survival (DSS), and progression-free survival (PFS) compared with Cluster B (**Figures 1D–F**). To detect the possible biological behavior, we screened a total of 3517 DEGs between SARGs-related molecular subtypes and identified that genes such as TAT, ALDH6A1, SCP2, SLC25A15, CAT, and PCK2 was highly expressed in Cluster A (**Figure 1G**). Then GO enrichment analysis of these DEGs showed that biological processes (BP) were primarily related to amino acid metabolism, while cellular components (CC) were associated with nuclear synaptic membranes, neuronal cell bodies, and apical plasma membranes and molecular functions (MF) were enriched in receptor-ligand activity, monoatomic ion channel activity and channel activity (**Figure 1H**). KEGG pathway enrichment analysis indicated that these genes were predominantly involved in amino acid metabolism, drug metabolism and fatty acid metabolism pathways (**Figure 1I**). We found that the pathways associated with tumor malignancy, such as G2M_CHECKPOINT, E2F_TARGETS, and MYC_TARGETS_V2, were upregulated in Cluster B by GSVA pathway analysis, potentially resulting in poorer prognosis (**Figure 1J**). Additionally, mutation analysis revealed that patients in Cluster B had a higher frequency of TP53 mutations compared with Cluster A, suggesting aggressive proliferation and invasion potential (**Figure 1K**).

### Immune infiltration and therapeutic sensitivity predictions in SARGs subtypes

**Figure 2:**
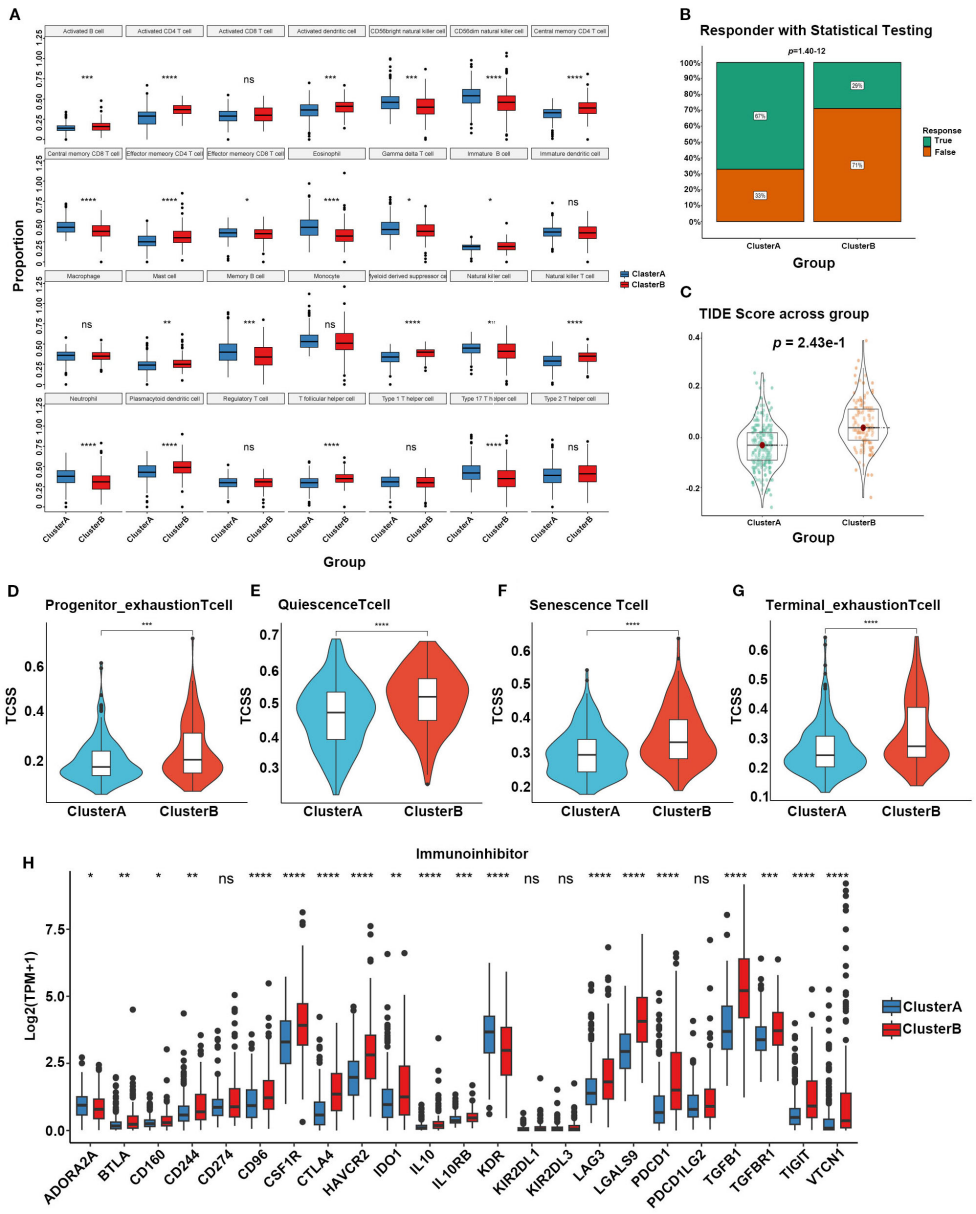
**(A)** Different contributions of immune cell infiltration in the two SARG subtypes based on ssGSEA algorithm. **(B)** Chi-square tests to confirm the differences of immunotherapy response between the two SARG subtypes. **(C)** TIDE scores of the two SARG subtypes. **(D–G)** T-cell status between the two SARG subtypes. **(H)** Expression levels of immune-suppressive regulatory factors in the two SARG subtypes (*p<0.05, **p<0.01, ***p<0.001, ****p<0.0001, ns: no significance).

We also assessed the potential roles of SARGs in the tumor microenvironment of HCC. Through ssGSEA algorithm, we analyzed the infiltration levels of 28 immune cell types. We found that CD56bright natural killer cells, CD56dim natural killer cells, central memory CD8 T cells, gamma delta T cells, immature B cells, memory B cells, natural killer cells, and neutrophils were more abundant in Cluster A (**Figure 2A**). Besides, Cluster A exhibited significantly lower TIDE scores, which were generally associated with reduced immune escape and a favorable immune microenvironment (**Figures 2B, C**) (20). We also evaluated T cell functional status between the two subtypes and found that patientsxin Cluster B exhibited higher levels of Progenitor_exhaustion, Quiescence, Senescence and Terminal_exhaustion status (**Figures 2D–G**), implying immune-suppressive microenvironment. We further analyzed the levels of immune-suppressive regulatory factors between subtypes and found the elevation in Cluster B, validating above findings. These factors may ultimately lead to a worse prognosis of Cluster B.

### Construction of a prognostic model for HCC based on SARGs

**Figure 3:**
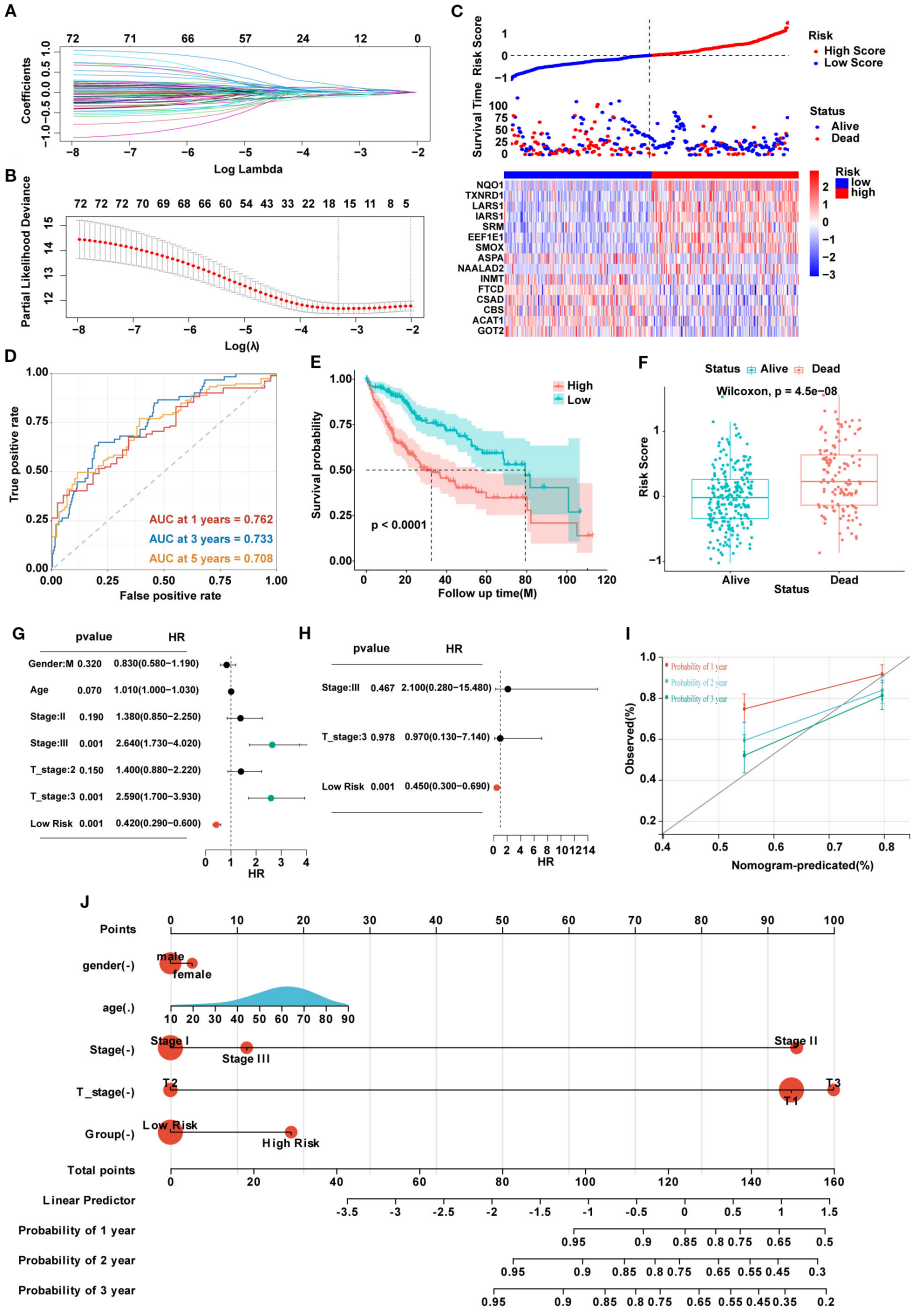
**(A)** Lasso coefficients of LASSO regression screening genes. **(B)** Identification of genes to model the prognostic risk score. **(C)** Ranked dot and scatter plots showing the risk score distribution and patient survival status, and the heatmap showing differential expression genes of the model in the high-risk and low-risk groups. **(D)** A time-dependent ROC curve to test the accuracy of the risk model. **(E, F)** The survival difference of patients with different risk patterns. **(G, H)** Univariate and multivariate analysis to confirm the independent prognosis function of the model. **(I)** A calibration curve to assess the accuracy of the model in predicting patients’ survival time. **(J)** The nomogram to obtain the predicting survival time of HCC patients.

The 81 SARGs were used to construct the risk model. We constructed the optimal prognostic signature by LASSO regression and conducted 10-fold cross-validation to narrow down the gene list (**Figures 3A, B**). Finally, 15 genes were included into the model, and the signature was constructed as follows: Risk score=(0.166×expression of LARS1)+(0.130×expression of TXNRD1)-(0.107×expression of GOT2)+(0.101×expression of NAALAD2)-(0.076×expression of CSAD)+(0.069×expression of SMOX)-(0.061×expression of ACAT1)-(0.058×expression of FTCD)+(0.039×expression of SRM)+(0.039×expression of IARS1)+(0.034×expression of EEF1E1)-(0.031×expression of INMT)-(0.022×expression of ASPA)-(0.016×expression of CBS)+(0.005×expression of NQO1). The patients were further divided into high- and low-risk group based on the median risk score (**Figure 3C**). We plotted ROC curve and demonstrate that the areas under the curve (AUROC) of the risk model to predict the prognosis in all patients with HCC were 0.762, 0.733, and 0.708 at 1, 3, and 5 years, respectively (**Figure 3D**). And the cohort with high-risk score was suggested to have worse prognosis (**Figures 3E, F**). Risk score were independent prognostic predictors according to univariate and multivariate COX regression analyses (**Figures 3G, H**). Then, we established the nomogram combining clinical feature to predict the 1-, 2-, and 3-year survival for HCC patients. The calibration curve indicated that the predicted probability of the nomogram was close to the actual probability (**Figures 3I, J**).

### Biological characteristics of HCC patients in different risk groups

**Figure 4:**
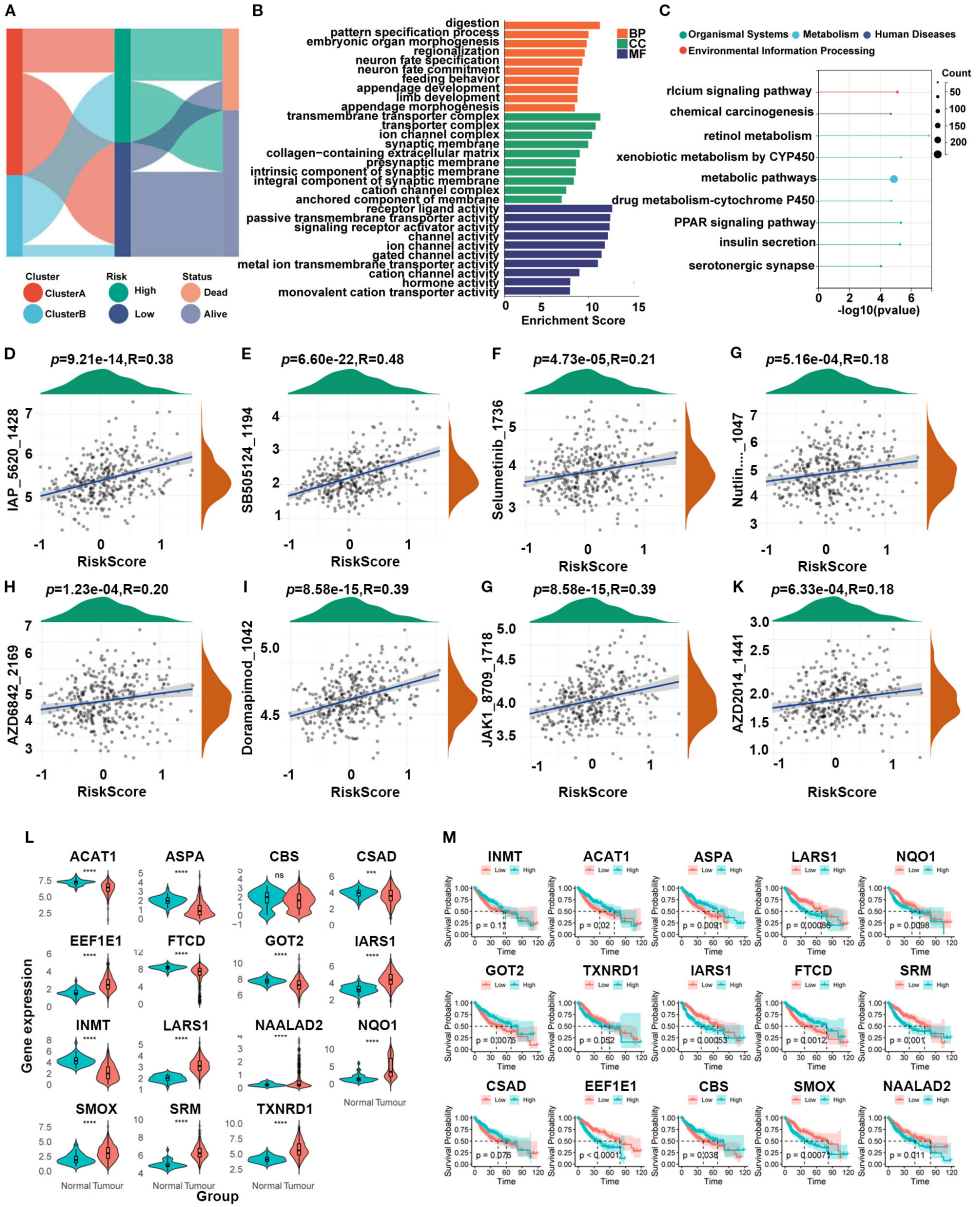
**(A)** Association between SARG subtypes, signature risk groups, and survival status. **(B, C)** GO and KEGG enrichment of differentially expressed genes between high- and low-risk subtypes. **(D–K)** Drug sensitivity based on GDSC database between the two risk groups. **(L)** Expression levels between tumor and adjacent non-tumor tissues of 15 model genes. **(M)** Gene-related prognosis of 15 model genes.

Sankey plots indicated a consistent relationship among the two SARGs molecular subtypes, the two signature risk groups and the prognosis of patients (**Figure 4A**). GO analysis revealed that the differences between the high- and low-risk group were mainly enriched in the activation of membrane channels and transmembrane transport of substances. KEGG enrichment analysis found that the metabolic pathways and drug metabolism of xenobiotics by cytochrome P450 pathways were highly enriched (**Figures 4B, C**). Cytochrome P450, as a superfamily of iron-ontaining heme proteins widely expressed in organisms, plays a pivotal role in detoxification, drug metabolism and regulation of endogenous substances (21). Meanwhile, its activity and genetic polymorphisms have crucial impact on drug treatment and clinical medication. Therefore, we conducted drug sensitivity analyses using the GDSC database and found that patients in low-risk group showed significant sensitivity to AZD2014 and JAK1 inhibitors (**Figures 4D–K**). To further find new therapeutic targets, we analyzed the expression and OS of 15 model genes and found that LARS1 was highly expressed in tumors and significantly associated with poor prognosis (**Figures 4L, M**). In addition, LARS1 also made the greatest contribution to the construction of the risk model with the highest coefficient. Therefore, we extracted LARS1 for the subsequent analysis.

### The high expression of LARS1 is associated with poor prognosis in HCC

**Figure 5:**
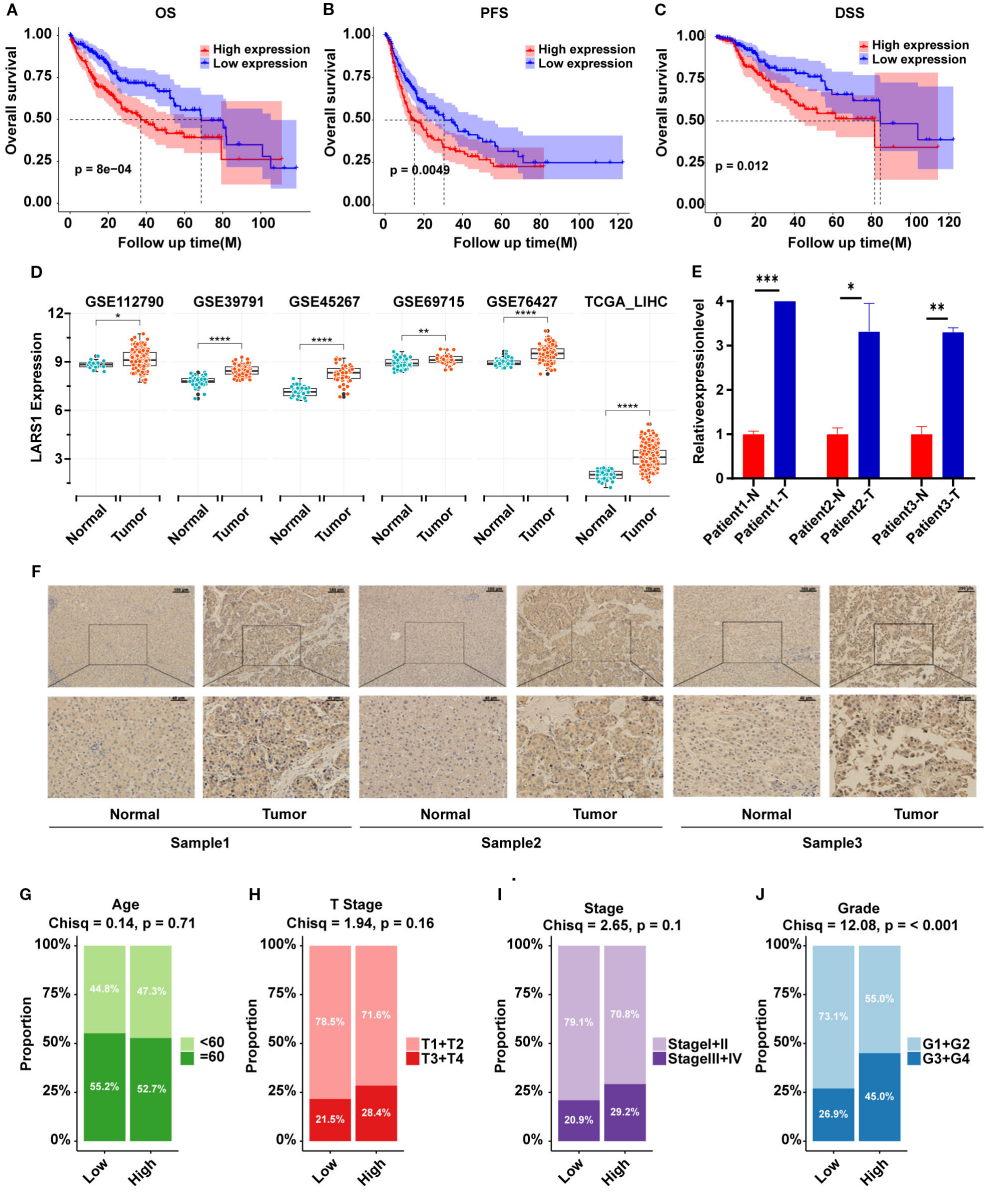
**(A–C)** OS, PFS and DSS plot between high- and low-LARS1 group based on TCGA. **(D)** The expression levels of LARS1 between tumor and adjacent non-tumor tissues from GEO database. **(E)** The mRNA levels of LARS1 between cancerous and adjacent non-tumor tissues based on PCR. **(F)** Representative pictures of IHC staining for LARS1 in cancerous and adjacent non-tumor tissues. **(G–J)** Chi-square test of clinical characteristics in high- and low-LARS1 groups based on TCGA (*p<0.05, **p<0.01, ***p<0.001, ****p<0.0001, ns: no significance).

To further explore the role of LARS1 in HCC, we investigated the prognosis of high- and low-LARS1, including OS, PFS and DSS based on TCGA. Results indicated that high LARS1 expression was associated with poorer prognosis of HCC patients (**Figures 5A–C**). Furthermore, LARS1 expression was higher in tumor tissues than in adjacent non-tumor tissues, which was verified in different datasets of GEO (**Figure 5D**). Moreover, to verify above findings, we detected LARS1 expression in 3 pairs of fresh and 50 pairs of paraffin-embedded HCC samples undergoing hepatectomy. The mRNA and protein levels of LARS1 were significantly higher in the cancer than those in adjacent non-tumor tissues based on PCR and IHC. (**Figures 5E, F**). This suggested that LARS1 might be a potential oncogene in HCC, which is consistent with the previous findings (16). We conducted further analysis to compare the clinical characteristics including age, gender, stage, grade and other related clinical parameters between high- and low-LARS1 groups based on TCGA. We found that patients with high-LARS1 levels tended to have significantly higher tumor grades (*P*<0.001), while patients also appeared to have more advanced stages in high-LARS1 group, although without statistical significance (*P*=0.1) (**Figures 5G–J**), suggesting the potential relationship between LARS1 and the differentiation grade of HCC. GO analysis indicated that DEGs between LARS1-related subgroups were mainly involved in metabolic-related biological processes and the binding to RNA or proteins based on TCGA cohort (**Supplementary Figure S2A**). In addition, we carried out an enrichment analysis of the pathways included in the Hallmark gene set through GSEA. We found that high LARS1 mainly associated with Myc targets v1, G2M checkpoint, E2F targets, Mitotic spindle, etc, related to the proliferation of tumor cells, dysregulation of the cell cycle, genomic instability, and drug resistance, indicating the occurrence and development of tumors (**Supplementary Figure S2B**).

### Single-cell RNA sequencing analysis of LARS1associated with amino acid metabolism in HCC

**Figure 6:**
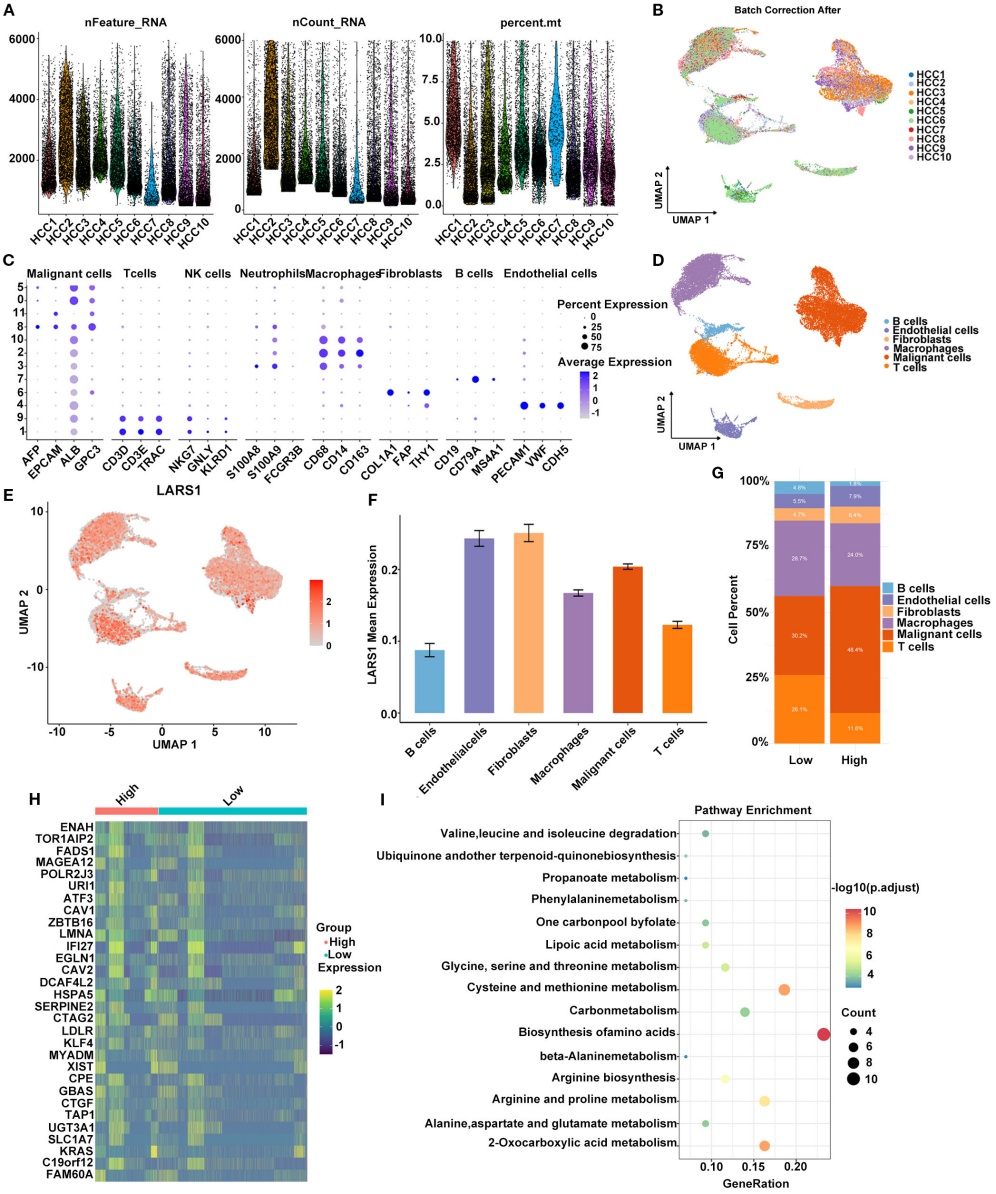
**(A)** Quality control metrics of the scRNA-seq data, including gene count, gene feature and mitochondrial gene percentage. **(B)** Batch effect correction across different HCC samples using Harmony integration. **(C)** Bubble plot showing the expression levels and proportions of marker genes across identified cell types. **(D)** Cell type annotation based on markers and clustering. **(E)** UMAP plot illustrating the expression pattern of LARS1 at the single-cell level. **(F)** Average expression level of LARS1 across different cell types. **(G)** Comparison of immune cell infiltration between high- and low-LARS1 groups. **(H)** Heatmap showing DEGs between high- and low-LARS1 malignant cells. **(I)** Pathway enrichment analysis of DEGs.

To further elucidate the potential role of LARS1 in HCC and its association with amino acid metabolism, we performed a comprehensive analysis based on single-cell RNA sequencing data. Specifically, we analyzed scRNA-seq data from multiple HCC samples in the GSE149614 dataset. After quality control and batch effect correction, cells were annotated into distinct types using canonical marker genes (**Figures 6A, B**). A bubble plot illustrated the expression of representative markers across clusters to ensure annotation accuracy (**Figure 6C**). Six major cell types were identified: B cells, endothelial cells, fibroblasts, macrophages, malignant cells, and T cells (**Figure 6D**). Next, we visualized the distribution of LARS1 expression across cell types using a UMAP plot and found that LARS1 was expressed in multiple cell populations (**Figure 6E**). Interestingly, LARS1 was not only highly expressed in malignant cells but also showed elevated expression in endothelial and fibroblast cells, suggesting that these non-malignant cells might also exhibit tumor-promoting activity (**Figure 6F**). To investigate the relationship between LARS1 expression and the tumor immune microenvironment, we divided malignant cells into LARS1-high and LARS1-low groups and evaluated differences in immune infiltration. The LARS1-low group exhibited a higher proportion of infiltrating immune cells (**Figure 6G**). We further conducted differential expression analysis between the two groups and visualized the top DEGs in a heatmap (**Figure 6H**).

Functional enrichment analysis of these DEGs revealed significant enrichment in pathways such as biosynthesis of amino acids, carbon metabolism, and cysteine and methionine metabolism, suggesting a potential role of LARS1 in regulating amino acid metabolic reprogramming in HCC (**Figure 6I**).

### Knockdown of LARS1 inhibits the proliferation, invasion and migration of hepatocellular carcinoma cells

**Figure 7:**
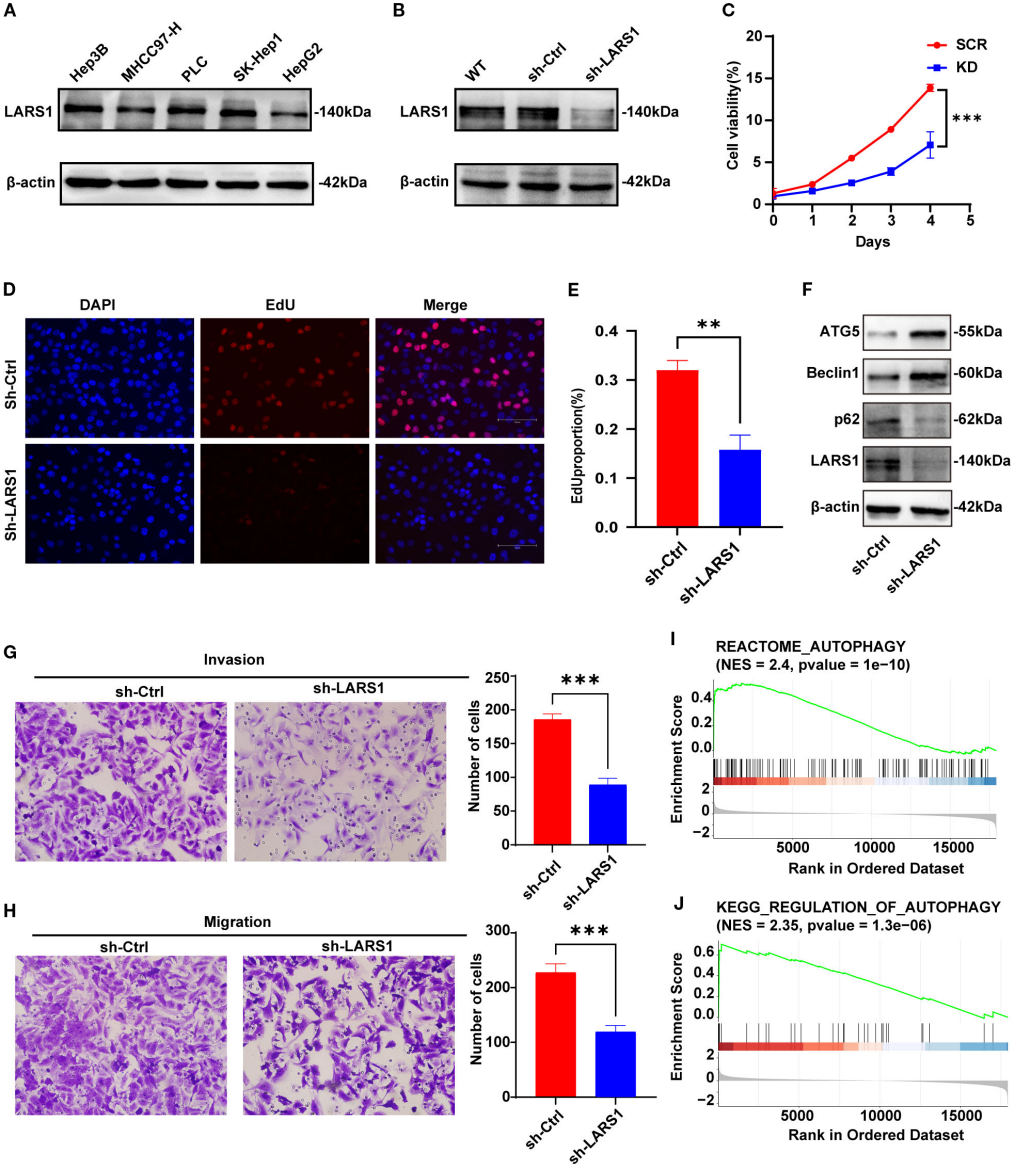
**(A)** Expression levels of LARS1 in different HCC cell lines by Western blotting. **(B)** Downregulation efficiency of LARS1 in SK-Hep1 validated by Western blotting. **(C)** Cell viability of SCR and KD groups. **(D, E)** Representative images and quantitative bar chart of EdU staining showing the effect of knockdown of LARS1 on the proliferation of SK-Hep1. **(F)** Western blot analysis was performed to detect autophagy-related protein levels. **(G, H)** Alteration in invasion and migration by knockdown of LARS1. **(I, J)** GSEA analysis of the correlation between LARS1 expression and autophagy related genes in TCGA cohort (**p<0.01, ***p<0.001).

Then, we explored the biological functions of LARS1 in the proliferation and metastasis of HCC. We initially assessed LARS1 expression levels in different HCC cell lines, including Hep3B, MHCC97-H, PLC, SK-Hep-1 and HepG2, and found that LARS1 expression was higher in the SK-Hep-1 cell line. As a result, we used SK-Hep-1 cells to establish stable LARS1 knockdown cell lines, and the efficiency of LARS1 deletion was validated by WB. (**Figures 7A, B**). We performed GSEA analysis based on TCGA and found that LARS1 expression was positively associated with cell cycle progression (**Supplementary Figure S2C**). CCK-8 and Edu assays revealed that suppression of LARS1 markedly inhibited the growth of HCC cells (**Figures 7C–E**). Additionally, our findings indicated that LARS1 knockdown significantly impeded cellular migration and invasion *in vitro* through Transwell chamber and Matrigel invasion assays (**Figures 7G, H**). Collectively, these results suggest that downregulation of LARS1 expression contributes to the inhibition of HCC cells. We further investigated the role of LARS1 in amino acid metabolism. KEGG analysis were conducted based on DEGs between high- and low-LARS1 groups and found the enrichment in autophagy and related pathways (**Supplementary Figure S2D**). Besides, the genesets of autophagy were also significantly enriched in low-LARS1 group according to GSEA analysis (**Figures 7I, J**). Previous studies have shown that amino acid deprivation can trigger autophagy by inhibition of mTORC1 to release free amino acids as part of a starvation adaptation (22). Additionally, glutamine- and autophagy-mediated restoration of mTORC1 in turn induces autophagy termination (23). Based on these findings with regard to autophagy in amino acid metabolism, we assessed the level of autophagy flux *in vitro*. Western blot revealed higher expression level of ATG5 and Beclin1 in LARS1 knockdown cells, along with reduced P62, indicating increased autophagy after knockdown of LARS1 (**Figure 7F**).

## Discussion

Cancer cells often encounter hypoxic and hypo-nutrient conditions for the huge consumption of metabolites (24). Therefore, metabolic alterations are recognized as hallmark of cancer. Metabolic reprogramming is a crucial process to sustain a favorable microenvironment to satisfy the requirements for energy, biosynthetic precursors and survival signals for rapid cell proliferation, invasion and metastasis of cancer (25). The Warburg effect, an example of metabolic reprogramming, is the phenomenon that cancer cells prefer to undergo glycolysis and produce lactate even in the presence of oxygen. Warburg effect provides many benefits to compete and share energy, which can promote the growth rate of cancer cells, including HCC (26, 27).

As the largest gland in human body, liver plays a critical role in metabolism. And the metabolism process of carbohydrates, lipids, proteins, hormones, bile, and exogenous diet in liver influence the development of various diseases vice versa. Expect for glucose metabolism, the alterations also contain increased fatty acid synthesis, amino acid metabolism, nucleotide production and so on (28). Amino acid metabolism is critical in HCC, not only to synthesize proteins but also to produce energy or to influence other biological behaviors of tumor (6). For example, reprogramming of tyrosine metabolism and poor prognosis in HCC was reported (29). Glutamine addiction was described to provide carbon source for the TCA cycle and essential nitrogen for purine and pyrimidine nucleotides in HCC, which are necessary for DNA and RNA biosynthesis (30). Elevated expression of glutaminase (GLS1), a key enzyme for glutaminolysis pathway, is correlated with poorer differentiation, lymphatic metastasis, advanced TNM stage, and worse prognosis in HCC (31). Besides, the correlation between amino acid metabolism and the immune cell microenvironment in HCC was demonstrated. Alterations in amino acid metabolism lead to changes in responses of immune cells in the tumor microenvironment, thereby promoting immune evasion in HCC (32). Consequently, it is necessary to explore the amino acid metabolic profile in HCC to better understand the pathogenic mechanisms of HCC.

In the present study, we analyzed the molecular patterns and clinical significance of amino acid metabolism in HCC. Patients with SARG cluster B showed poorer prognosis and higher activity of pathways associated with malignancy progression, including G2M_CHECKPOINT, E2F_TARGETS, MYC_TARGETS_V2. Besides, the rate of mutation in TP53 in Cluster B was greatest and significantly higher compared with Cluster A. As we know, TP53 mutation contributes to carcinogenesis and tumor development, suggesting enhanced proliferative and invasive potential of Cluster B. In addition, immune cell infiltration also differed significantly between the two subtypes. Higher levels of infiltration of anti-tumor effector cells and lower TIDE scores were found in the Cluster A, indicating that patients in the Cluster A might be more sensitive to immune checkpoint inhibitors. Cluster B exhibited higher levels of Progenitor Exhaustion, Quiescence, Senescence, and Terminal Exhaustion according to the evaluation of functional status of T cells, indicating immunosuppressive subtype in Cluster B, which was corroborated by subsequent analysis of immunosuppressive regulators. Consequently, the classification based on amino acid metabolism revealed heterogeneity and immune microenvironment of HCC, which were anticipated to provide potential evidence for precision treatment.

Additionally, we screened 15 genes for the construction of the risk models by LASSO regression based on metabolism-related genes. There were significant differences in prognosis between the high- and low-risk groups. And ROC curves confirmed robust predictive performance of the risk model in distinguishing high- and low-risk HCC patients. The model genes were involved in multiple biological processes pertaining to HCC development. For instance, TXNRD1 and NQO1, as redox stress-related enzymes, have been reported to be upregulated in various cancers and promote malignant progression (33–35). GOT2 and FTCD are both key enzymes in amino acid metabolism, playing a critical role in maintaining hepatic amino acid balance. It is also demonstrated that silencing GOT2 reprograms glutamine metabolism, which increased the sensitivity to glutaminase inhibitors of HCC cells (36). We also established a nomogram for better clinical applicability by combining the risk score with clinical characteristics to further improve the performance of the risk signature. And the risk score was found to be independent risk factors for HCC patients by multivariate analyses after adjusting other variables, highlighting its potential clinical utility. It is worth noting that the risk model was only validated internally using TCGA and lacks validation from an external independent cohort. As a result, future studies are needed to validate the risk model for its prognostic prediction ability.

In addition, we also found that patients in the low-risk group were more sensitive to drugs such as AZD2014. AZD2014 is a dual inhibitor of mTORC1 and C2. It is demonstrated that AZD2014 resulted in more profound proliferation suppression, apoptosis, cell cycle arrest, and autophagy in HCC cells compared with rapamycin (37). Our study suggests that AZD2014 may influence amino acid metabolism via mTORC1. However, further investigation is required to confirm this in future studies. It is worth noting that targeting a single gene or metabolic enzyme is often insufficient to achieve an effective and safe anti-cancer effect in clinical trials, due to the high degree of heterogeneity of HCC (38). Consequently, the development of multi-target therapeutic strategies may represent a more promising alternative. For example, plant-derived products and extracts, composed by a blend of biologically active secondary metabolites, were demonstrated to modulate key metabolic processes through multi-target effect in HCC (39).

To deeply understand the mechanisms behind amino acid metabolism in HCC, further investigation of key genes in the model is needed. Among the 15-gene risk models, LARS1 has the highest risk coefficient and is most likely the key gene in the model. Previous study has suggested that LARS1 was an important oncogene in HCC and was associated with immune infiltration, consistent with our findings (16). We further knocked down the expression of LARS1 gene by shRNA in HCC cell lines, and demonstrated that knockdown of LARS1 inhibits the proliferation and migration of human HCC cells. However, only 3 pairs of tissues were included in our study, which might limit the statistical power of the results. Consequently, larger sample sizes are needed to confirm our findings in the future studies. Furthermore, we performed single-cell RNA sequencing analysis, and found that DEGs between high- and low-LARS1 group enriched in pathways including biosynthesis of amino acids, carbon metabolism, and cysteine and methionine metabolism, suggesting a potential role of LARS1 in regulating amino acid metabolic reprogramming in HCC. Single-cell transcriptomic analysis manifested the heterogeneity of LARS1 in HCC and suggested that it may contribute to tumor progression through modulation of amino acid metabolism.

Amino acid imbalance is observed in chronic liver disease including cirrhosis and related disease states, characterized by a reduction in BCAAs (including valine, leucine and isoleucine) and an elevation in aromatic amino acids (AAAs) in serum (40). It is demonstrated that reducing or removing BCAAs significantly suppressed the proliferation rates of the HCC cell line *in vitro* and high dietary BCAA intake enhances tumor development and growth *in vivo* of mouse models (10). Noteworthily, the underlying mechanism of BCAA promoting tumor development and growth was demonstrated to be the activation of mTORC1 signaling pathway by accumulation of BCAA, especially leucine, in HCC tumors. Leucine, as the major component of BCAA, induce the lysosomal localization of mTORC1 and its subsequent activation. And LARS1 was elaborated to be a key mediator for amino acid signaling to mTORC1. Lysosomal localization of mTORC1 was not observed and amino acid induced S6K phosphorylationin was inhibited in LARS1 knockdown cells. It is reported that LARS1, function as a leucine sensor and GTPase-activating protein (GAP), interacts with RagD GTPase for mTORC1 signaling by sensing intracellular leucine concentration (41, 42). mTORC1 regulates multiple biological process including autophagy (43). Autophagy is an evolutionarily conserved program that is responsible for degradation of dysfunctional or damaged organelles in all living cells, playing a crucial role in liver homeostasis (44). In cancer cells, autophagy is considered a double-edged sword, with tumor-suppressing features during tumorigenesis stage and then tumor-promoting properties after tumorigenesis. Autophagy was investigated to be activated in LARS1-downregulated cells (42). Besides, it is reported that branched-chain amino acid transaminase 1 (BCAT1) decreases cisplatin sensitivity of HCC by inducing mTOR-mediated autophagy via leucine metabolism *in vivo* and vitro (45). To further explore the molecular mechanism of LARS1 on amino acid metabolism in HCC, we conducted the Western blot of autophagy flux and found the increased autophagy in LARS1 knockdown cells. Consequently, we hypothesized that LARS1 might impede autophagy via regulating amino acid metabolism to activate the mTORC1 pathway, thereby impacting HCC progression, which provided novel insights compared with previous studies. As a result, LARS1 might be a potential valuable biomarker and molecular therapeutic target for HCC therapy.

This study has several limitations. Firstly, the risk model based on 15 genes was validated solely using an TCGA datasets via retrospective analyses. Thus, external validation in future studies are needed to further validate the risk model. Secondly, the role of LARS1 in amino acid metabolism of HCC warrants systematic experimental verification. Additionally, direct experimental evidence for LARS1 regulating mTORC1 downstream proteins, such as p-S6K/p-4EBP1, is currently lacking in our study, the molecular mechanism of LARS1 in regulating mTORC1 and autophagy needs to be further studied both *in vivo* and *in vitro* in the future studies.

In conclusion, we have successfully developed a prognostic model based on 15 genes associated with amino acid metabolism and identified LARS1 as the key gene. Moreover, we revealed the heterogeneous expression of LARS1 in HCC and its potential tumor-promoting mechanisms by single-cell transcriptomic analysis. Finally, we verified that knockdown of LARS1 significantly inhibited the proliferation, invasion and migration of HCC *in vitro*, with increased autophagy flux, indicating that LARS1 could be a potential therapeutic target for HCC.

## Data Availability

The datasets presented in this study can be found in online repositories. The names of the repository/repositories and accession number(s) can be found in the article/**Supplementary Material**.
