# Supplementary material for: Identification of amino acid metabolism-related gene Leucyl-tRNA synthetase 1 (LARS1) as a potential prognostic and therapeutic target in hepatocellular carcinoma
Source: Front Oncol. 2025 Sep 16;15:1675018. doi: 10.3389/fonc.2025.1675018 (PMC12479282; doi:10.3389/fonc.2025.1675018)
Supplement: Supplementary file 1 [file DataSheet1.zip › 原始数据/In vitro experiment/PCR/LARS1_1.pdf]

Monitoring - AmplifyData

All colors

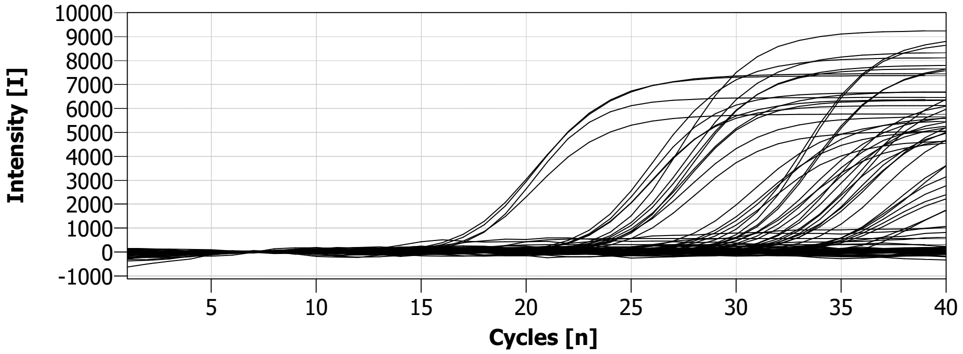

FAM

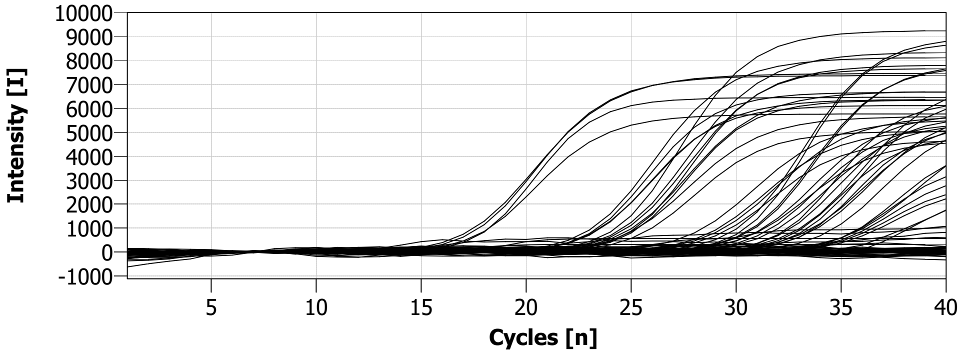

Monitoring - MeltingData

All colors

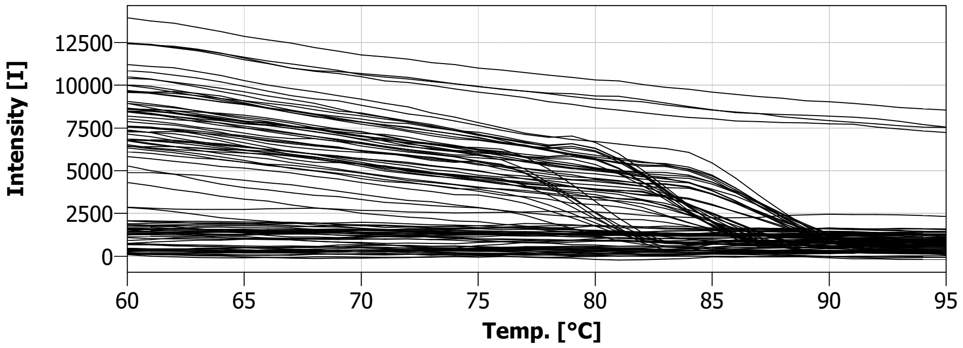

FAM

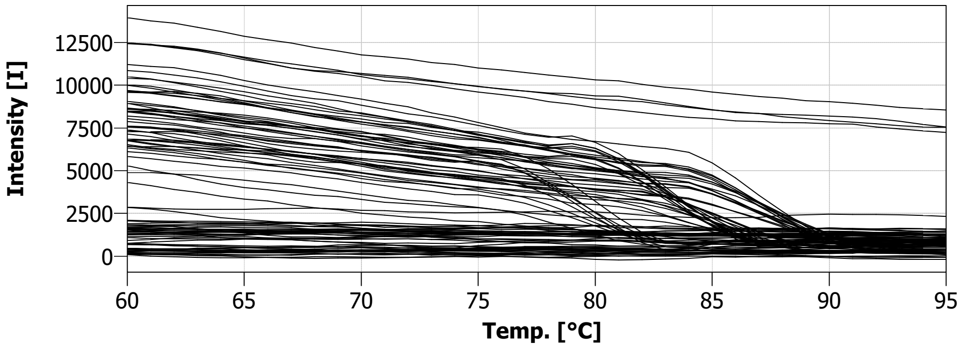

Ct

CT

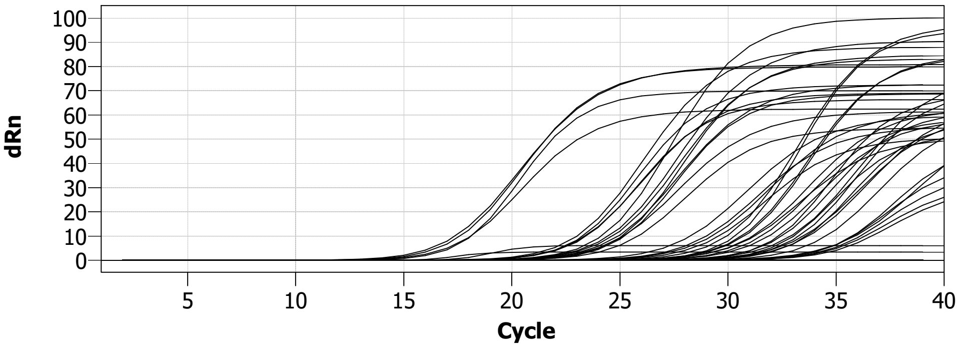

Ct

Well		Ct	Sample name	Sample type	Dye	Gene	Mean Ct
A1	[redacted]	No Ct		Unknown	FAM		
A2		No Ct		Unknown	FAM		
A3		31.5		Unknown	FAM		31.5
A4		30.87		Unknown	FAM		30.87
A5		30.16		Unknown	FAM		30.16
A6		30.52		Unknown	FAM		30.52
A7		24.29		Unknown	FAM		24.29
A8		23.95		Unknown	FAM		23.95
A9		24.81		Unknown	FAM		24.81
A10		24.12		Unknown	FAM		24.12
A11		No Ct		Unknown	FAM		
A12		No Ct		Unknown	FAM		
B1		No Ct		Unknown	FAM		
B2		No Ct		Unknown	FAM		
B3		No Ct		Unknown	FAM		
B4		No Ct		Unknown	FAM		
B5		35.06		Unknown	FAM		35.06
B6		33.5		Unknown	FAM		33.5
B7		31.88		Unknown	FAM		31.88
B8		32.68		Unknown	FAM		32.68
B9		32.64		Unknown	FAM		32.64
B10		32.31		Unknown	FAM		32.31
B11		No Ct		Unknown	FAM		
B12		No Ct		Unknown	FAM		
C1		No Ct		Unknown	FAM		
C2		No Ct		Unknown	FAM		
C3		28.17		Unknown	FAM		28.17
C4		28.49		Unknown	FAM		28.49
C5		27.96		Unknown	FAM		27.96
C6		27.19		Unknown	FAM		27.19
C7		16.69		Unknown	FAM		16.69
C8		16.38		Unknown	FAM		16.38
C9		17.16		Unknown	FAM		17.16
C10		17.28		Unknown	FAM		17.28
C11		No Ct		Unknown	FAM		
C12		No Ct		Unknown	FAM		
D1		No Ct		Unknown	FAM		
D2		No Ct		Unknown	FAM		
D3		No Ct		Unknown	FAM		
D4		34.7		Unknown	FAM		34.7
D5		35.23		Unknown	FAM		35.23

Ct

Well		Ct	Sample name	Sample type	Dye	Gene	Mean Ct
D6	[redacted]	35.49		Unknown	FAM		35.49
D7		22.38		Unknown	FAM		22.38
D8		22.07		Unknown	FAM		22.07
D9		22.48		Unknown	FAM		22.48
D10		22.35		Unknown	FAM		22.35
D11		No Ct		Unknown	FAM		
D12		No Ct		Unknown	FAM		
E1		No Ct		Unknown	FAM		
E2		No Ct		Unknown	FAM		
E3		32.11		Unknown	FAM		32.11
E4		31.43		Unknown	FAM		31.43
E5		32.39		Unknown	FAM		32.39
E6		30.11		Unknown	FAM		30.11
E7		No Ct		Unknown	FAM		
E8		No Ct		Unknown	FAM		
E9		No Ct		Unknown	FAM		
E10		No Ct		Unknown	FAM		
E11		No Ct		Unknown	FAM		
E12		No Ct		Unknown	FAM		
F1		No Ct		Unknown	FAM		
F2		No Ct		Unknown	FAM		
F3		34.77		Unknown	FAM		34.77
F4		34.76		Unknown	FAM		34.76
F5		21.19		Unknown	FAM		21.19
F6		No Ct		Unknown	FAM		
F7		No Ct		Unknown	FAM		
F8		No Ct		Unknown	FAM		
F9		No Ct		Unknown	FAM		
F10		No Ct		Unknown	FAM		
F11		No Ct		Unknown	FAM		
F12		No Ct		Unknown	FAM		
G1		No Ct		Unknown	FAM		
G2		No Ct		Unknown	FAM		
G3		23.48		Unknown	FAM		23.48
G4		23.23		Unknown	FAM		23.23
G5		23.66		Unknown	FAM		23.66
G6		24.17		Unknown	FAM		24.17
G7		No Ct		Unknown	FAM		
G8		No Ct		Unknown	FAM		
G9		No Ct		Unknown	FAM		
G10		No Ct		Unknown	FAM		

Ct

Well		Ct	Sample name	Sample type	Dye	Gene	Mean Ct
G11	[redacted]	No Ct		Unknown	FAM		
G12	[redacted]	No Ct		Unknown	FAM		
H1	[redacted]	No Ct		Unknown	FAM		
H2	[redacted]	No Ct		Unknown	FAM		
H3	[redacted]	29.44		Unknown	FAM		29.44
H4	[redacted]	29.03		Unknown	FAM		29.03
H5	[redacted]	29.67		Unknown	FAM		29.67
H6	[redacted]	29.8		Unknown	FAM		29.8
H7	[redacted]	No Ct		Unknown	FAM		
H8	[redacted]	No Ct		Unknown	FAM		
H9	[redacted]	No Ct		Unknown	FAM		
H10	[redacted]	No Ct		Unknown	FAM		
H11	[redacted]	No Ct		Unknown	FAM		
H12	[redacted]	No Ct		Unknown	FAM		

Tm

DIF - , Threshold: 0

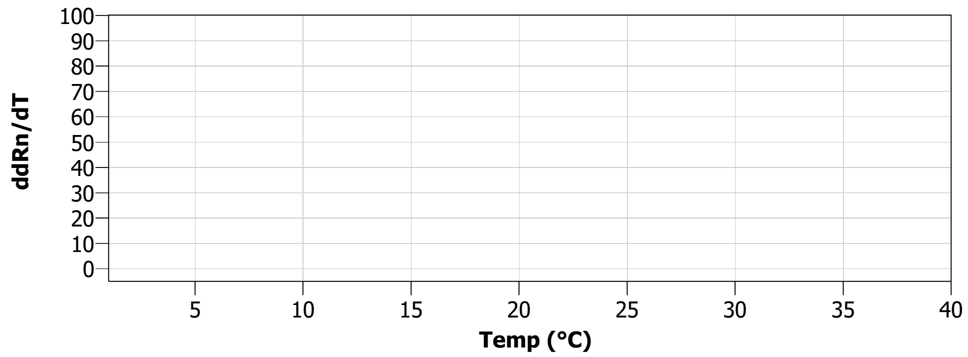

GOI -

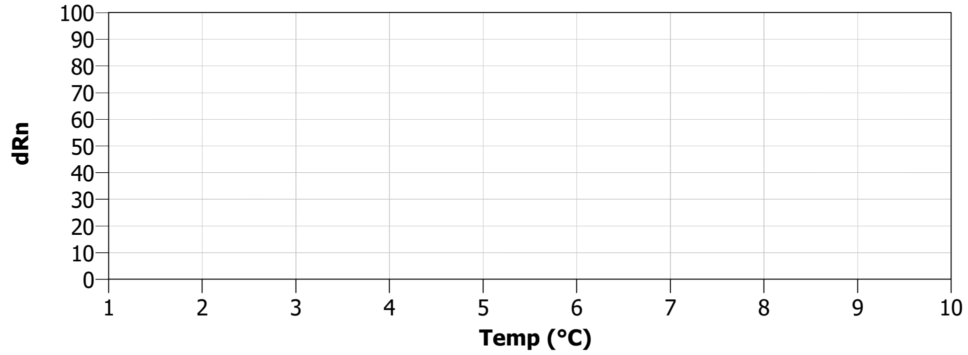

Tm

Well		Sample name	Sample type	Tm	Mean Tm	Std.Dev. Mean Tm
A1						
B1						
C1						
D1						
E1						
F1						
G1						
H1						
A2						
B2						
C2						
D2						
E2						
F2						
G2						
H2						
A3						
B3						
C3						
D3						
E3						
F3						
G3						
H3						
A4						
B4						
C4						
D4						
E4						
F4						
G4						
H4						
A5						
B5						
C5						
D5						
E5						
F5						
G5						
H5						
A6						

Tm

Well		Sample name	Sample type	Tm	Mean Tm	Std.Dev. Mean Tm
B6	[redacted]					
C6	[redacted]					
D6	[redacted]					
E6	[redacted]					
F6	[redacted]					
G6	[redacted]					
H6	[redacted]					
A7	[redacted]					
B7	[redacted]					
C7	[redacted]					
D7	[redacted]					
E7	[redacted]					
F7	[redacted]					
G7	[redacted]					
H7	[redacted]					
A8	[redacted]					
B8	[redacted]					
C8	[redacted]					
D8	[redacted]					
E8	[redacted]					
F8	[redacted]					
G8	[redacted]					
H8	[redacted]					
A9	[redacted]					
B9	[redacted]					
C9	[redacted]					
D9	[redacted]					
E9	[redacted]					
F9	[redacted]					
G9	[redacted]					
H9	[redacted]					
A10	[redacted]					
B10	[redacted]					
C10	[redacted]					
D10	[redacted]					
E10	[redacted]					
F10	[redacted]					
G10	[redacted]					
H10	[redacted]					
A11	[redacted]					
B11	[redacted]					

Tm

Well		Sample name	Sample type	Tm	Mean Tm	Std.Dev. Mean Tm
C11	[redacted]					
D11	[redacted]					
E11	[redacted]					
F11	[redacted]					
G11	[redacted]					
H11	[redacted]					
A12	[redacted]					
B12	[redacted]					
C12	[redacted]					
D12	[redacted]					
E12	[redacted]					
F12	[redacted]					
G12	[redacted]					
H12	[redacted]					
