# Supplementary material for: Identification of amino acid metabolism-related gene Leucyl-tRNA synthetase 1 (LARS1) as a potential prognostic and therapeutic target in hepatocellular carcinoma
Source: Front Oncol. 2025 Sep 16;15:1675018. doi: 10.3389/fonc.2025.1675018 (PMC12479282; doi:10.3389/fonc.2025.1675018)
Supplement: Supplementary file 1 [file DataSheet1.zip › 原始数据/In vitro experiment/PCR/LARS1_2.pdf]

Monitoring - AmplifyData

All colors

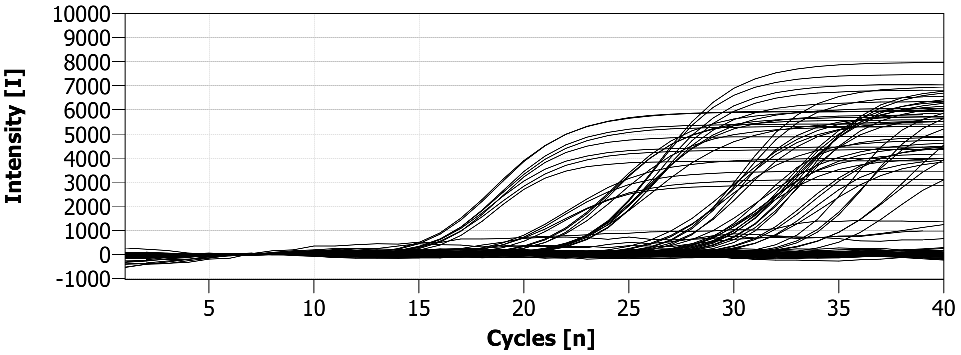

FAM

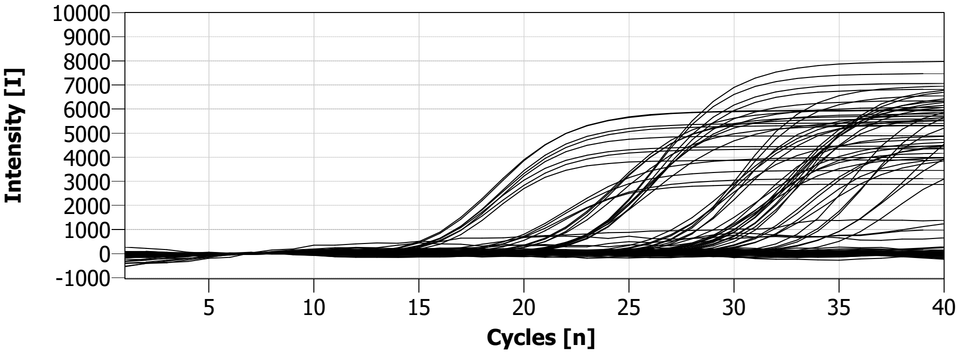

Monitoring - MeltingData

All colors

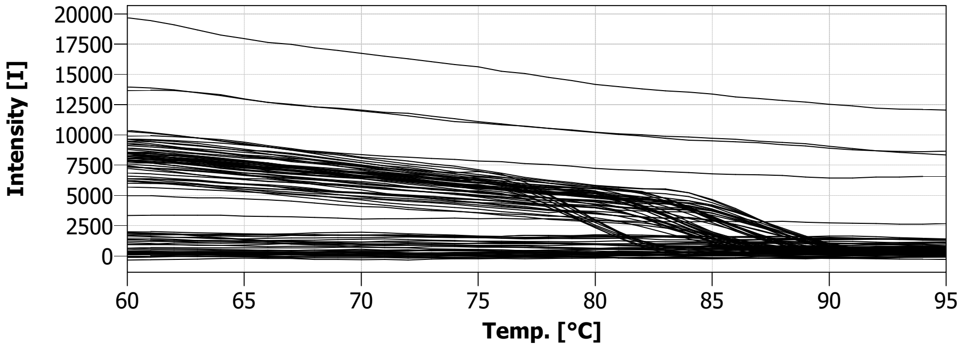

FAM

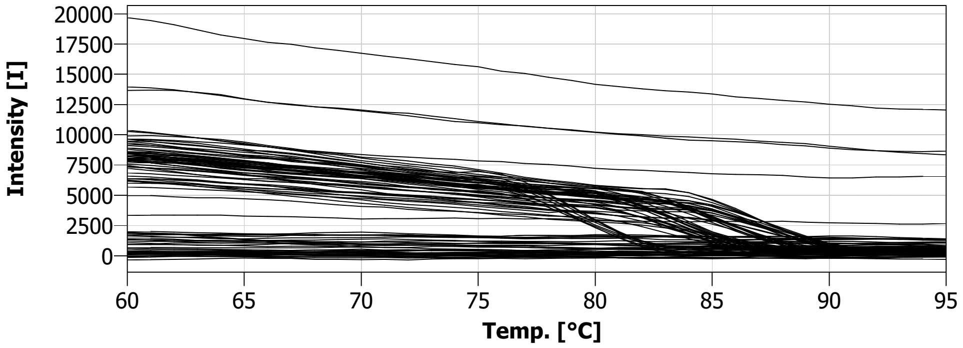

Ct

CT

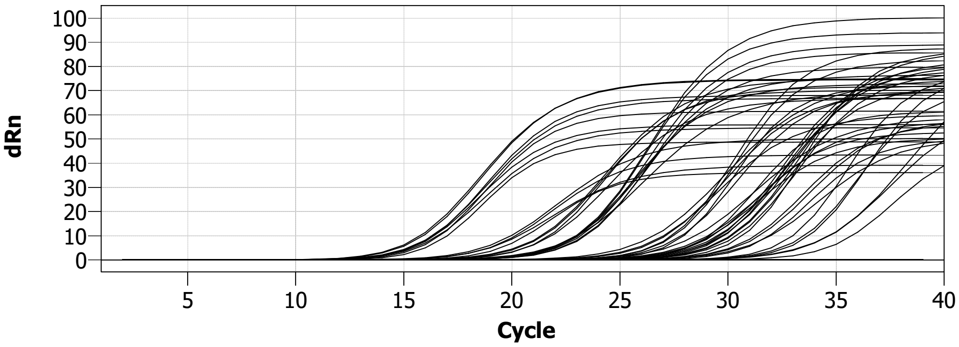

Ct

Well		Ct	Sample name	Sample type	Dye	Gene	Mean Ct
A1	[redacted]	No Ct		Unknown	FAM		
A2		No Ct		Unknown	FAM		
A3		19.04		Unknown	FAM		19.04
A4		19.26		Unknown	FAM		19.26
A5		19.33		Unknown	FAM		19.33
A6		19.79		Unknown	FAM		19.79
A7		28.34		Unknown	FAM		28.34
A8		28.42		Unknown	FAM		28.42
A9		28.14		Unknown	FAM		28.14
A10		28.31		Unknown	FAM		28.31
A11		No Ct		Unknown	FAM		
A12		No Ct		Unknown	FAM		
B1		No Ct		Unknown	FAM		
B2		No Ct		Unknown	FAM		
B3		16.25		Unknown	FAM		16.25
B4		15.61		Unknown	FAM		15.61
B5		15.78		Unknown	FAM		15.78
B6		15		Unknown	FAM		15
B7		22.16		Unknown	FAM		22.16
B8		22.09		Unknown	FAM		22.09
B9		22.52		Unknown	FAM		22.52
B10		22.13		Unknown	FAM		22.13
B11		No Ct		Unknown	FAM		
B12		No Ct		Unknown	FAM		
C1		No Ct		Unknown	FAM		
C2		No Ct		Unknown	FAM		
C3		27.06		Unknown	FAM		27.06
C4		26.72		Unknown	FAM		26.72
C5		27.23		Unknown	FAM		27.23
C6		26.62		Unknown	FAM		26.62
C7		30.42		Unknown	FAM		30.42
C8		31.11		Unknown	FAM		31.11
C9		30.58		Unknown	FAM		30.58
C10		31.05		Unknown	FAM		31.05
C11		No Ct		Unknown	FAM		
C12		No Ct		Unknown	FAM		
D1		No Ct		Unknown	FAM		
D2		No Ct		Unknown	FAM		
D3		22.6		Unknown	FAM		22.6
D4		22.27		Unknown	FAM		22.27
D5		22.36		Unknown	FAM		22.36

Ct

Well		Ct	Sample name	Sample type	Dye	Gene	Mean Ct
D6	[redacted]	22.16		Unknown	FAM		22.16
D7	[redacted]	25.6		Unknown	FAM		25.6
D8	[redacted]	26.12		Unknown	FAM		26.12
D9	[redacted]	26.25		Unknown	FAM		26.25
D10	[redacted]	27.53		Unknown	FAM		27.53
D11	[redacted]	No Ct		Unknown	FAM		
D12	[redacted]	No Ct		Unknown	FAM		
E1	[redacted]	No Ct		Unknown	FAM		
E2	[redacted]	No Ct		Unknown	FAM		
E3	[redacted]	No Ct		Unknown	FAM		
E4	[redacted]	34.92		Unknown	FAM		34.92
E5	[redacted]	33.76		Unknown	FAM		33.76
E6	[redacted]	No Ct		Unknown	FAM		
E7	[redacted]	20.97		Unknown	FAM		20.97
E8	[redacted]	21		Unknown	FAM		21
E9	[redacted]	20.49		Unknown	FAM		20.49
E10	[redacted]	20.61		Unknown	FAM		20.61
E11	[redacted]	No Ct		Unknown	FAM		
E12	[redacted]	No Ct		Unknown	FAM		
F1	[redacted]	No Ct		Unknown	FAM		
F2	[redacted]	No Ct		Unknown	FAM		
F3	[redacted]	29.06		Unknown	FAM		29.06
F4	[redacted]	28.83		Unknown	FAM		28.83
F5	[redacted]	28.66		Unknown	FAM		28.66
F6	[redacted]	29.97		Unknown	FAM		29.97
F7	[redacted]	15.11		Unknown	FAM		15.11
F8	[redacted]	15.5		Unknown	FAM		15.5
F9	[redacted]	15.57		Unknown	FAM		15.57
F10	[redacted]	15.96		Unknown	FAM		15.96
F11	[redacted]	No Ct		Unknown	FAM		
F12	[redacted]	No Ct		Unknown	FAM		
G1	[redacted]	No Ct		Unknown	FAM		
G2	[redacted]	No Ct		Unknown	FAM		
G3	[redacted]	32.75		Unknown	FAM		32.75
G4	[redacted]	33.69		Unknown	FAM		33.69
G5	[redacted]	33.02		Unknown	FAM		33.02
G6	[redacted]	32.24		Unknown	FAM		32.24
G7	[redacted]	No Ct		Unknown	FAM		
G8	[redacted]	No Ct		Unknown	FAM		
G9	[redacted]	No Ct		Unknown	FAM		
G10	[redacted]	No Ct		Unknown	FAM		

Ct

Well		Ct	Sample name	Sample type	Dye	Gene	Mean Ct
G11	[redacted]	No Ct		Unknown	FAM		
G12	[redacted]	No Ct		Unknown	FAM		
H1	[redacted]	No Ct		Unknown	FAM		
H2	[redacted]	No Ct		Unknown	FAM		
H3	[redacted]	29.56		Unknown	FAM		29.56
H4	[redacted]	29.35		Unknown	FAM		29.35
H5	[redacted]	28.92		Unknown	FAM		28.92
H6	[redacted]	29.27		Unknown	FAM		29.27
H7	[redacted]	No Ct		Unknown	FAM		
H8	[redacted]	No Ct		Unknown	FAM		
H9	[redacted]	No Ct		Unknown	FAM		
H10	[redacted]	No Ct		Unknown	FAM		
H11	[redacted]	No Ct		Unknown	FAM		
H12	[redacted]	No Ct		Unknown	FAM		

Tm

DIF - , Threshold: 0

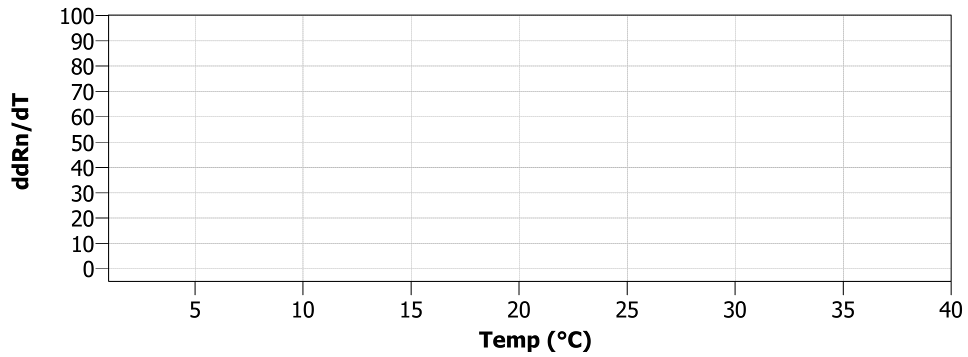

GOI -

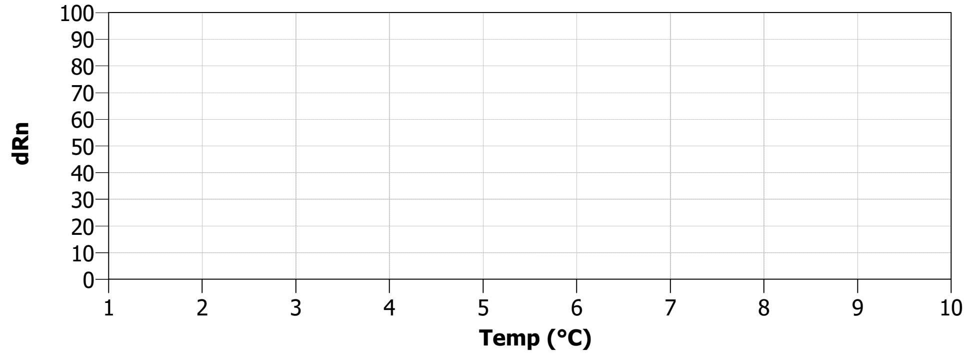

Tm

Well		Sample name	Sample type	Tm	Mean Tm	Std.Dev. Mean Tm
A1	[redacted]					
B1	[redacted]					
C1	[redacted]					
D1	[redacted]					
E1	[redacted]					
F1	[redacted]					
G1	[redacted]					
H1	[redacted]					
A2	[redacted]					
B2	[redacted]					
C2	[redacted]					
D2	[redacted]					
E2	[redacted]					
F2	[redacted]					
G2	[redacted]					
H2	[redacted]					
A3	[redacted]					
B3	[redacted]					
C3	[redacted]					
D3	[redacted]					
E3	[redacted]					
F3	[redacted]					
G3	[redacted]					
H3	[redacted]					
A4	[redacted]					
B4	[redacted]					
C4	[redacted]					
D4	[redacted]					
E4	[redacted]					
F4	[redacted]					
G4	[redacted]					
H4	[redacted]					
A5	[redacted]					
B5	[redacted]					
C5	[redacted]					
D5	[redacted]					
E5	[redacted]					
F5	[redacted]					
G5	[redacted]					
H5	[redacted]					
A6	[redacted]					

Tm

Well		Sample name	Sample type	Tm	Mean Tm	Std.Dev. Mean Tm
B6	[redacted]					
C6	[redacted]					
D6	[redacted]					
E6	[redacted]					
F6	[redacted]					
G6	[redacted]					
H6	[redacted]					
A7	[redacted]					
B7	[redacted]					
C7	[redacted]					
D7	[redacted]					
E7	[redacted]					
F7	[redacted]					
G7	[redacted]					
H7	[redacted]					
A8	[redacted]					
B8	[redacted]					
C8	[redacted]					
D8	[redacted]					
E8	[redacted]					
F8	[redacted]					
G8	[redacted]					
H8	[redacted]					
A9	[redacted]					
B9	[redacted]					
C9	[redacted]					
D9	[redacted]					
E9	[redacted]					
F9	[redacted]					
G9	[redacted]					
H9	[redacted]					
A10	[redacted]					
B10	[redacted]					
C10	[redacted]					
D10	[redacted]					
E10	[redacted]					
F10	[redacted]					
G10	[redacted]					
H10	[redacted]					
A11	[redacted]					
B11	[redacted]					

Tm

Well		Sample name	Sample type	Tm	Mean Tm	Std.Dev. Mean T[illegible]
C11	[redacted]					
D11	[redacted]					
E11	[redacted]					
F11	[redacted]					
G11	[redacted]					
H11	[redacted]					
A12	[redacted]					
B12	[redacted]					
C12	[redacted]					
D12	[redacted]					
E12	[redacted]					
F12	[redacted]					
G12	[redacted]					
H12	[redacted]					
