# Supplementary figures and images for: Identification of amino acid metabolism-related gene Leucyl-tRNA synthetase 1 (LARS1) as a potential prognostic and therapeutic target in hepatocellular carcinoma
Source: Front Oncol. 2025 Sep 16;15:1675018. doi: 10.3389/fonc.2025.1675018 (PMC12479282; doi:10.3389/fonc.2025.1675018)

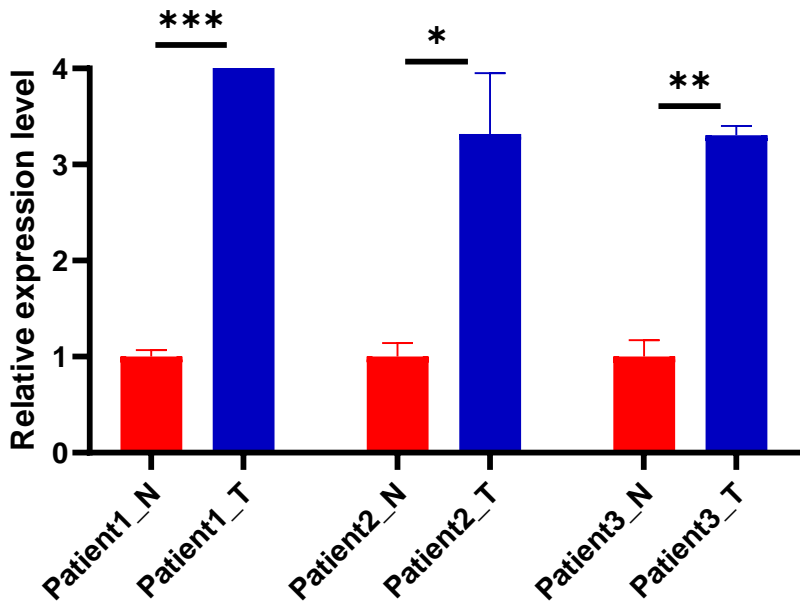

Supplement: Supplementary file 1 [file DataSheet1.zip › 原始数据/In vitro experiment/PCR/LARS1in tumor and adjcent.pdf]

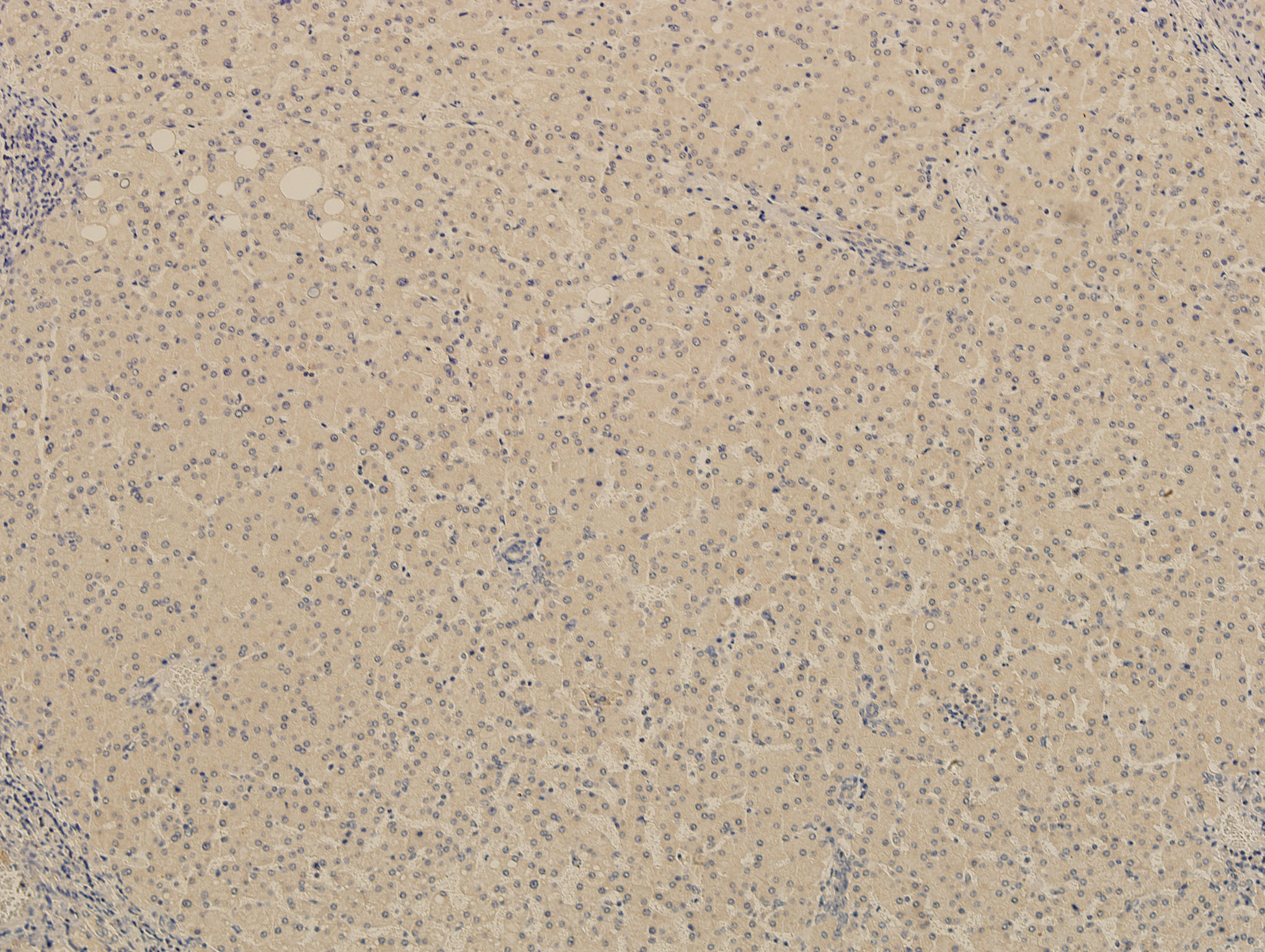

Supplement: Supplementary file 2 [file DataSheet2.zip › IHC/Sample1/2304417-12-10X-1.jpg]

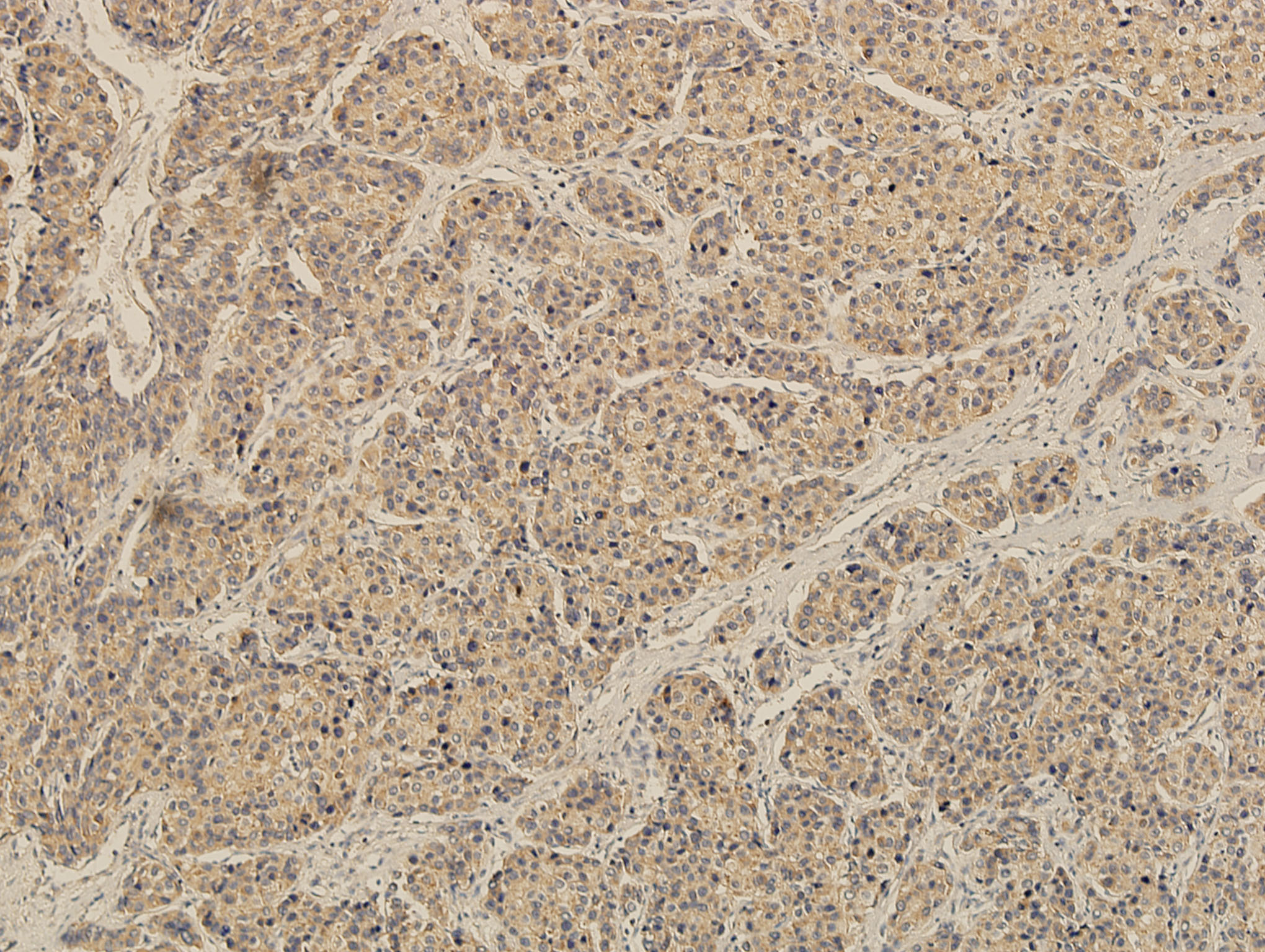

Supplement: Supplementary file 2 [file DataSheet2.zip › IHC/Sample1/2304417-8-10X-3.jpg]

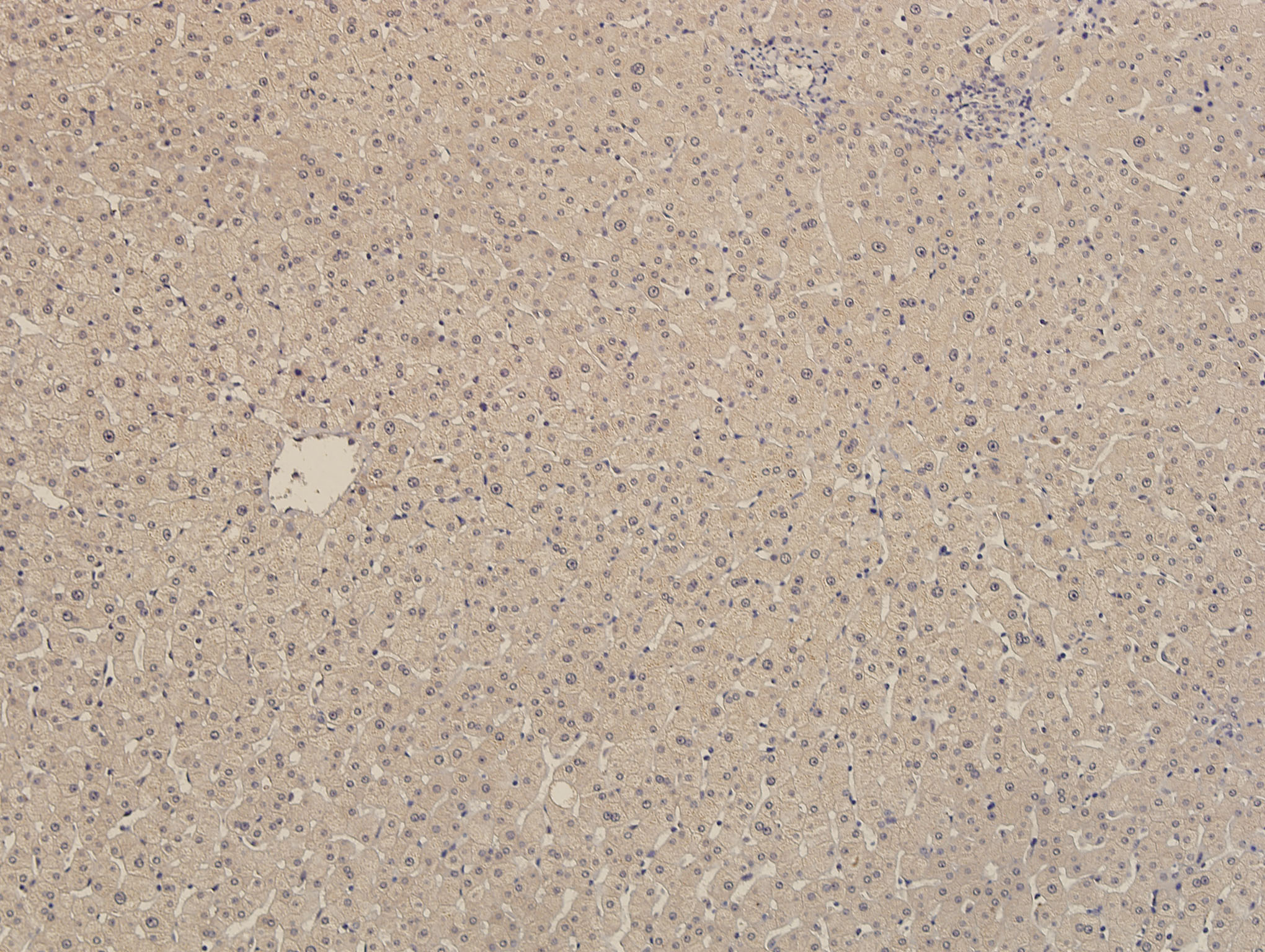

Supplement: Supplementary file 2 [file DataSheet2.zip › IHC/Sample2/2400713-10-10X-1.jpg]

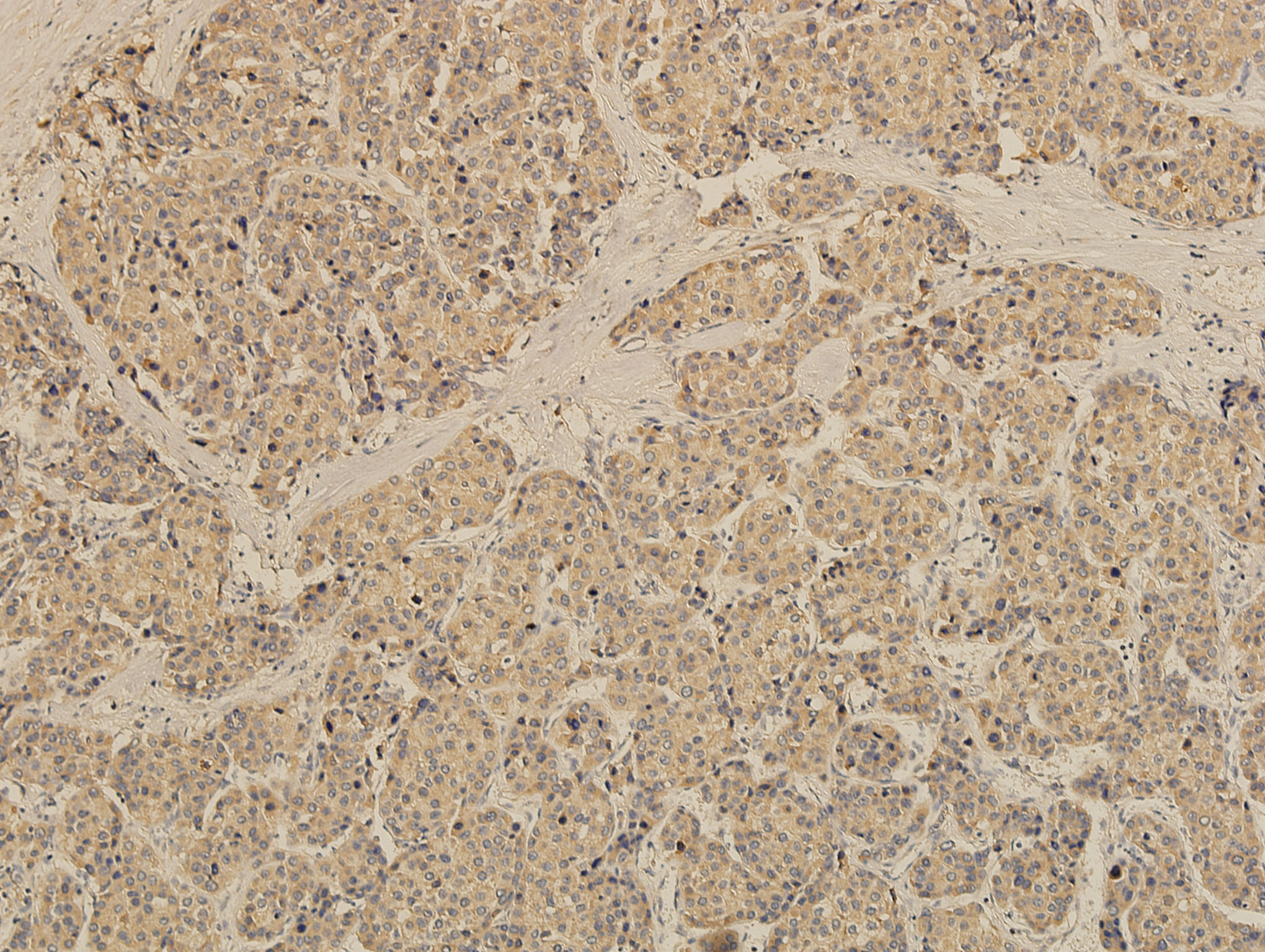

Supplement: Supplementary file 2 [file DataSheet2.zip › IHC/Sample2/2400713-3-10X-1.jpg]

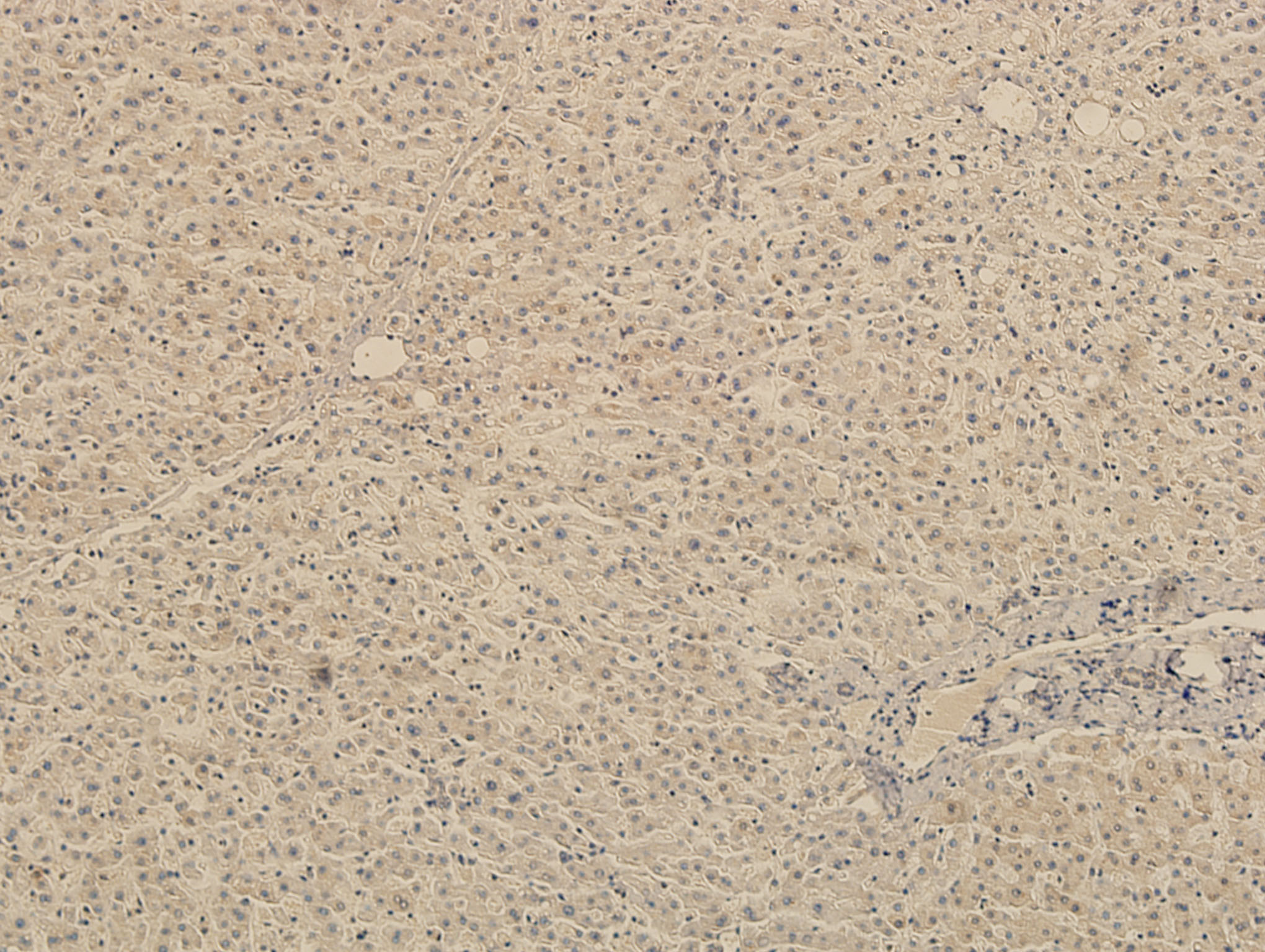

Supplement: Supplementary file 2 [file DataSheet2.zip › IHC/Sample3/2402147-11-10x-2.jpg]

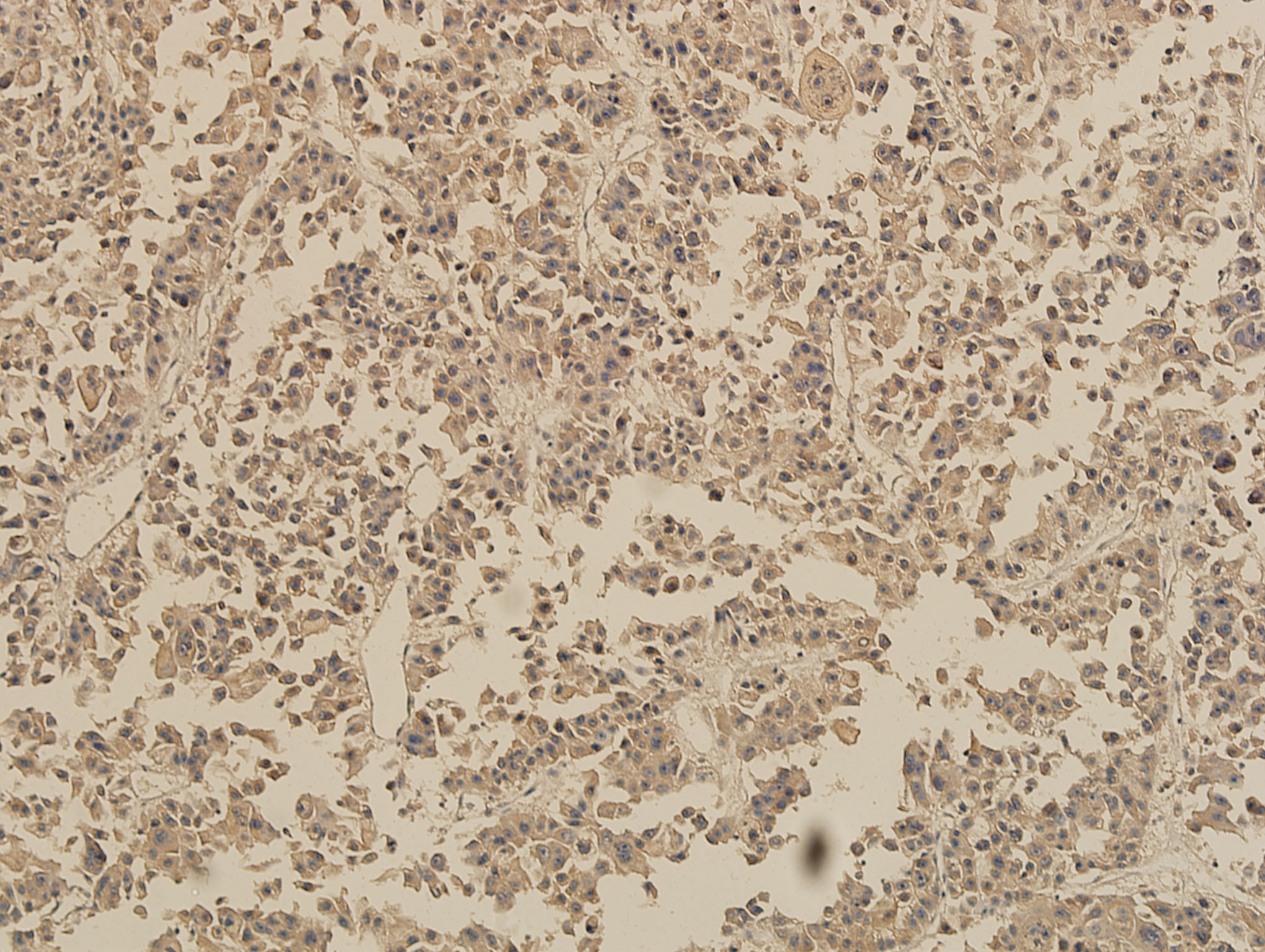

Supplement: Supplementary file 2 [file DataSheet2.zip › IHC/Sample3/2402147-4-10x-1.jpg]

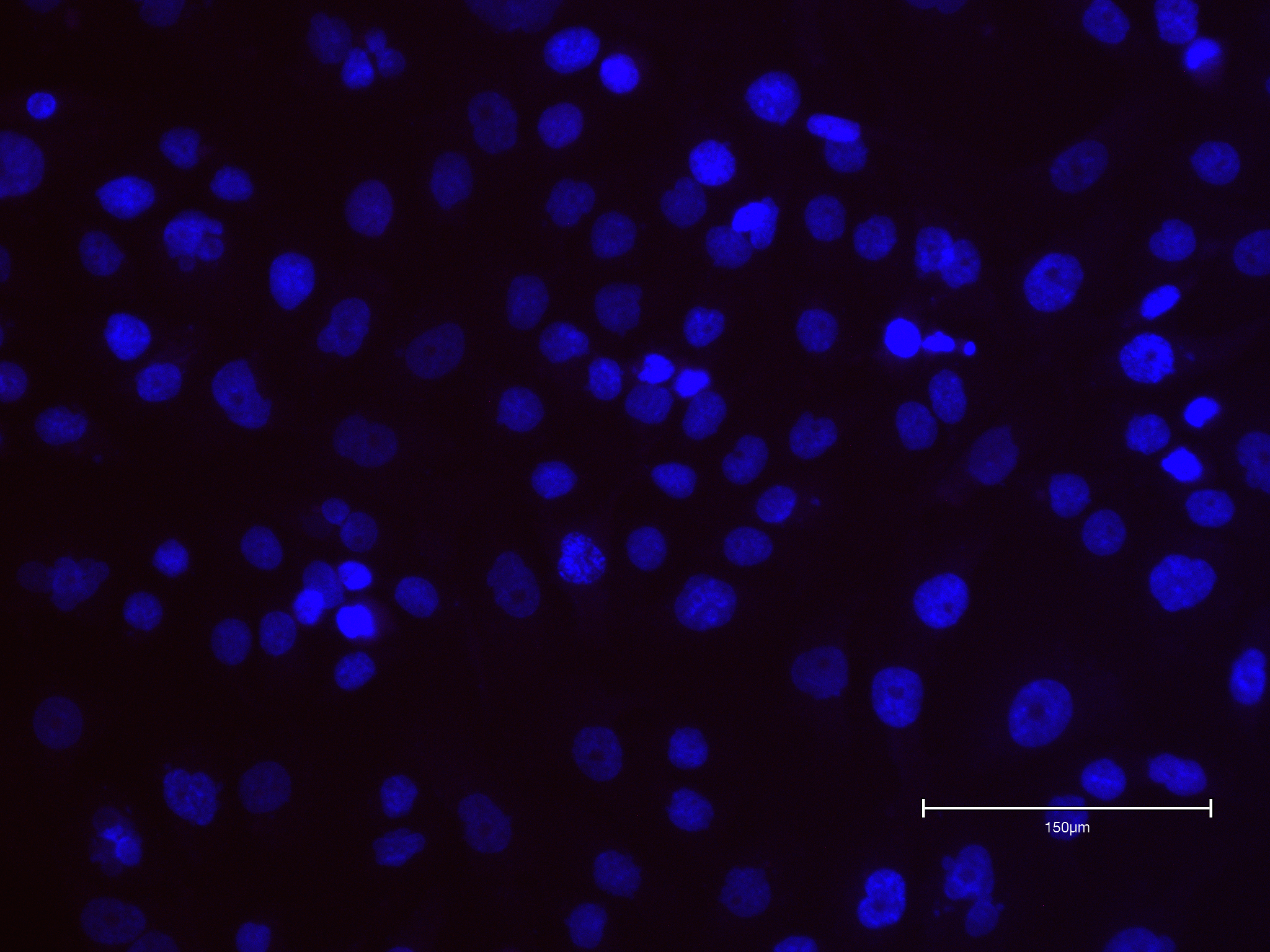

Supplement: Supplementary file 3 [file DataSheet3.zip › EDU/KD/SK-KD_0006.tif]

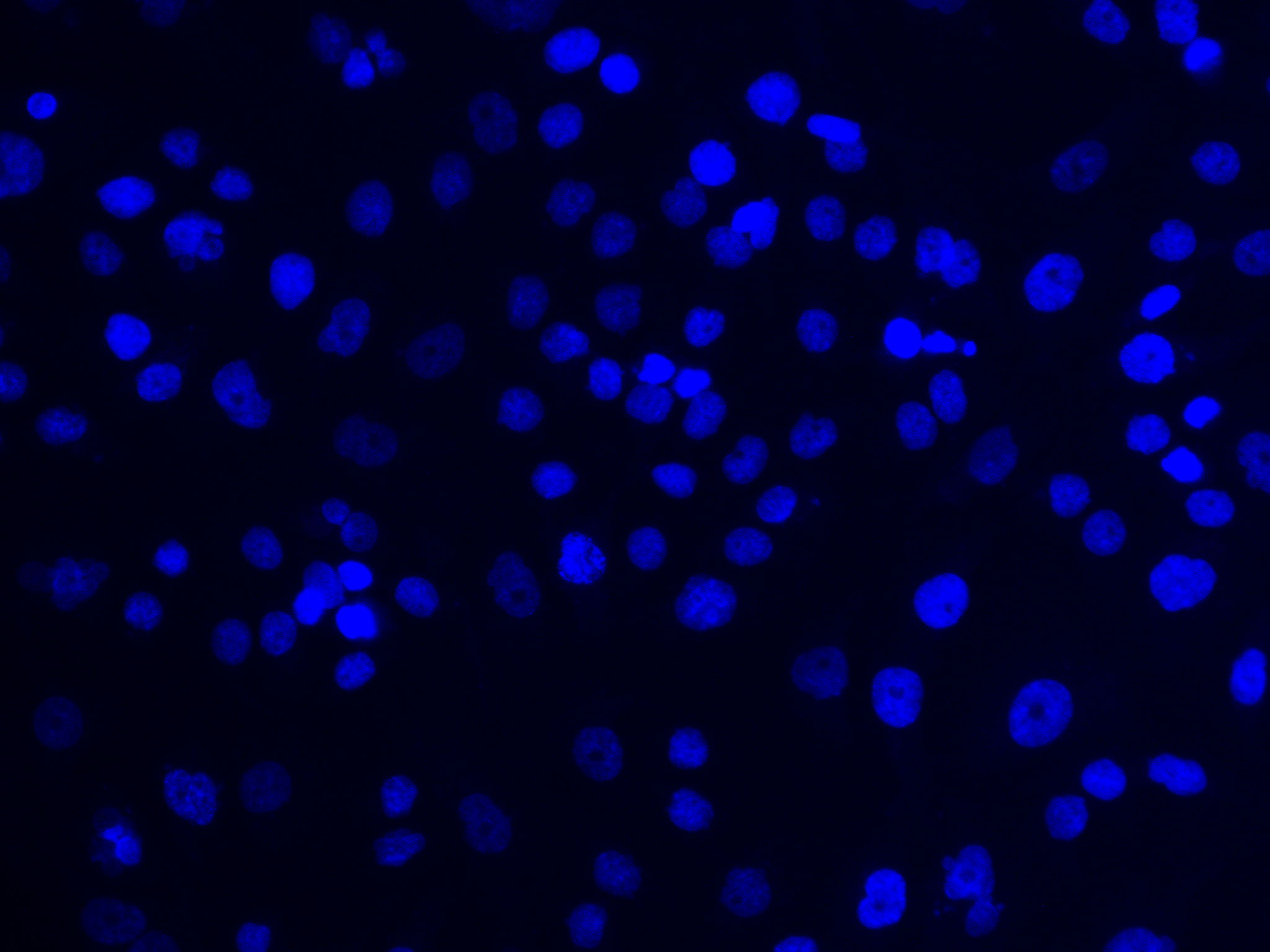

Supplement: Supplementary file 3 [file DataSheet3.zip › EDU/KD/SK-KD_0006_DAPI.tif]

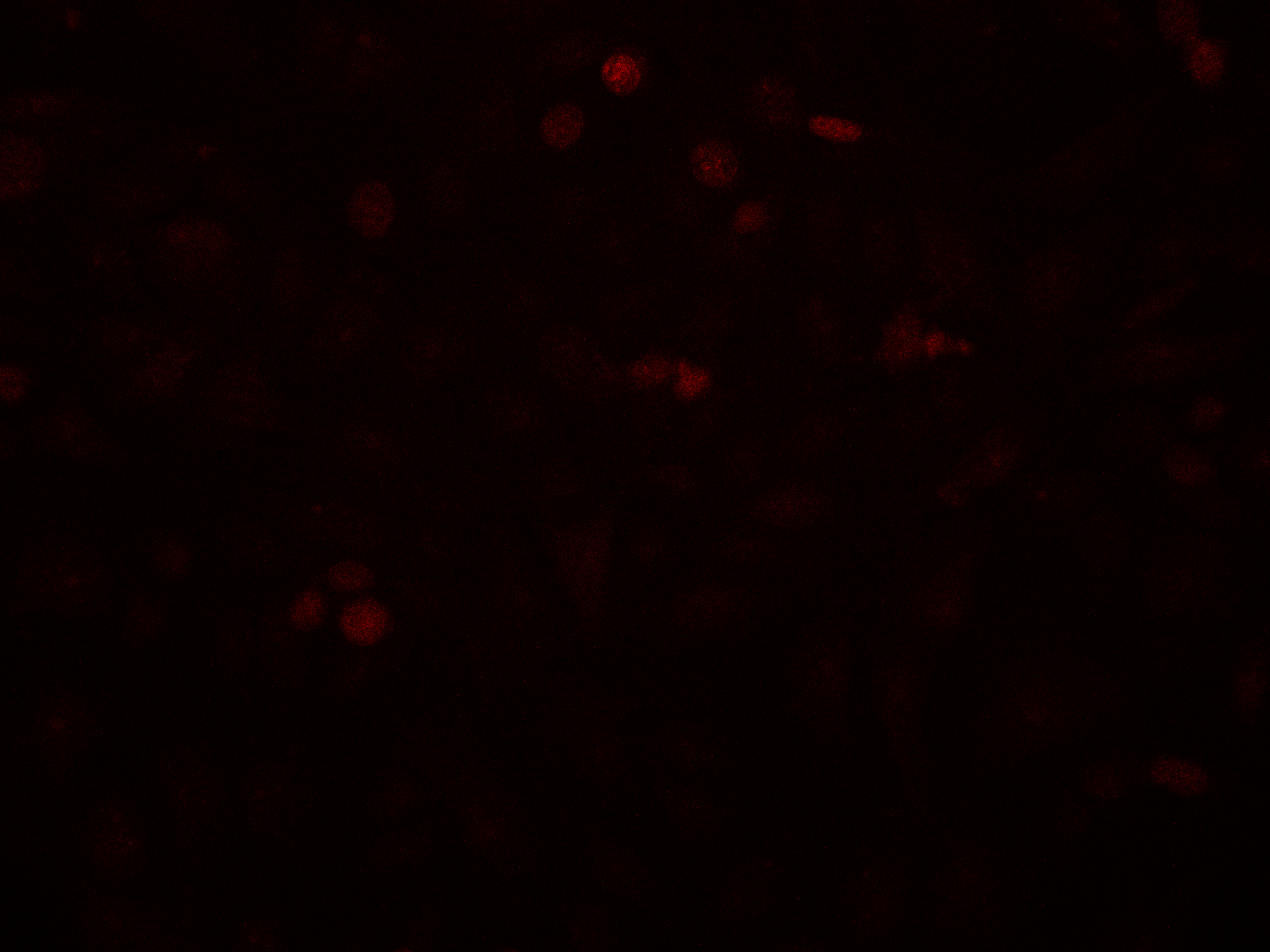

Supplement: Supplementary file 3 [file DataSheet3.zip › EDU/KD/SK-KD_0006_RFP.tif]

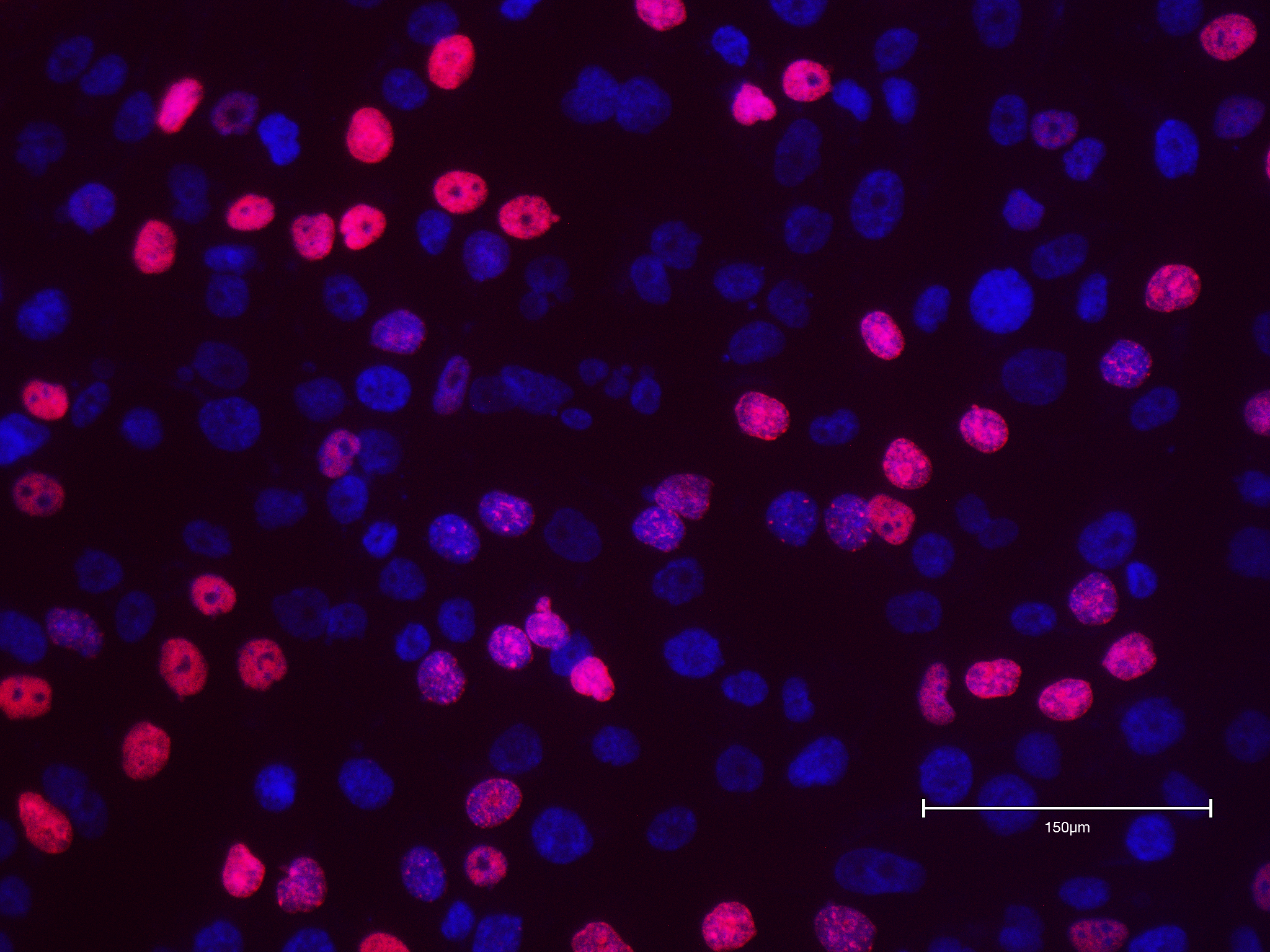

Supplement: Supplementary file 3 [file DataSheet3.zip › EDU/SCR/SK-SCR_0002.tif]

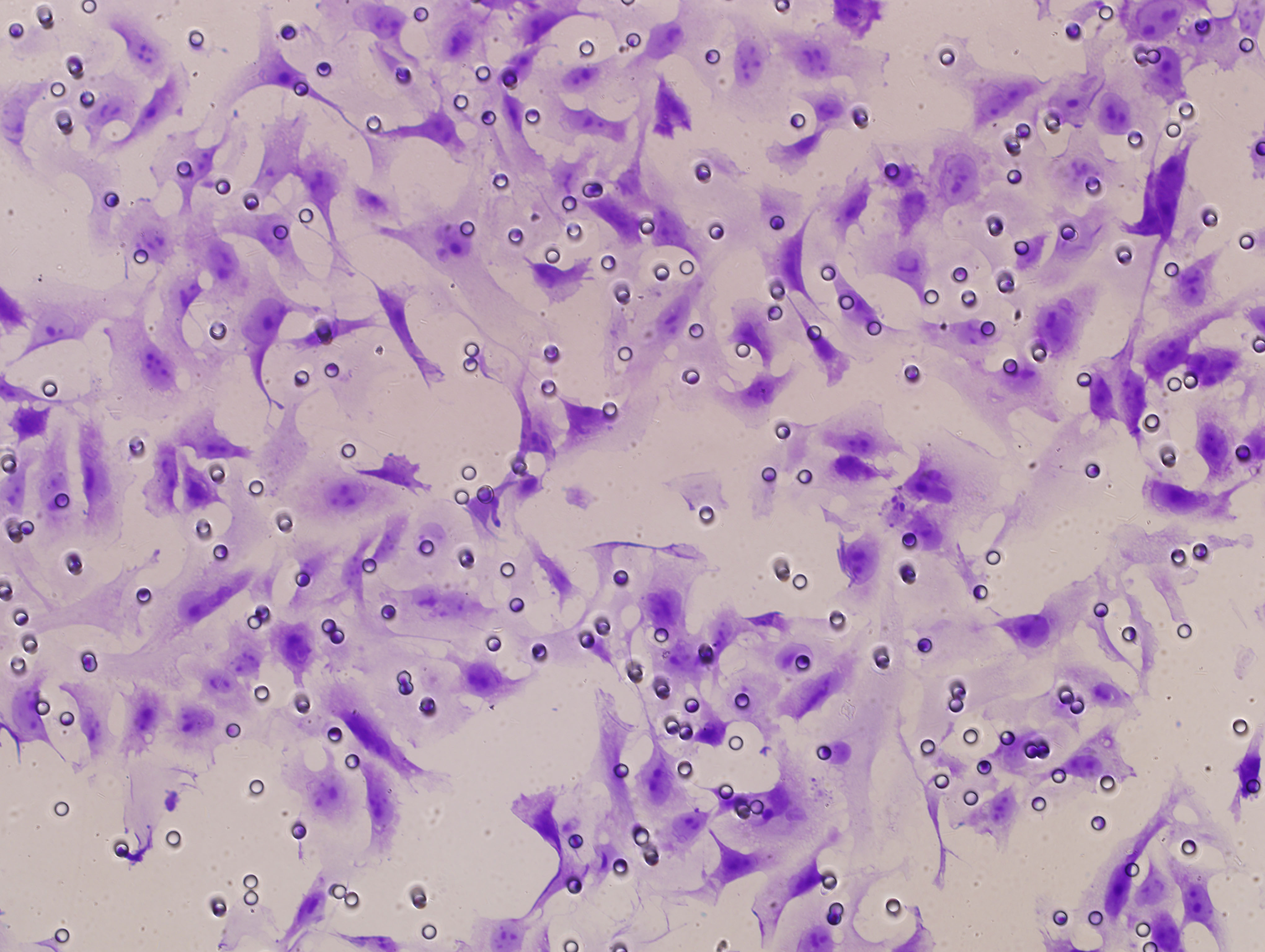

Supplement: Supplementary file 4 [file DataSheet4.zip › Invasion/Invasion Kd.jpg]

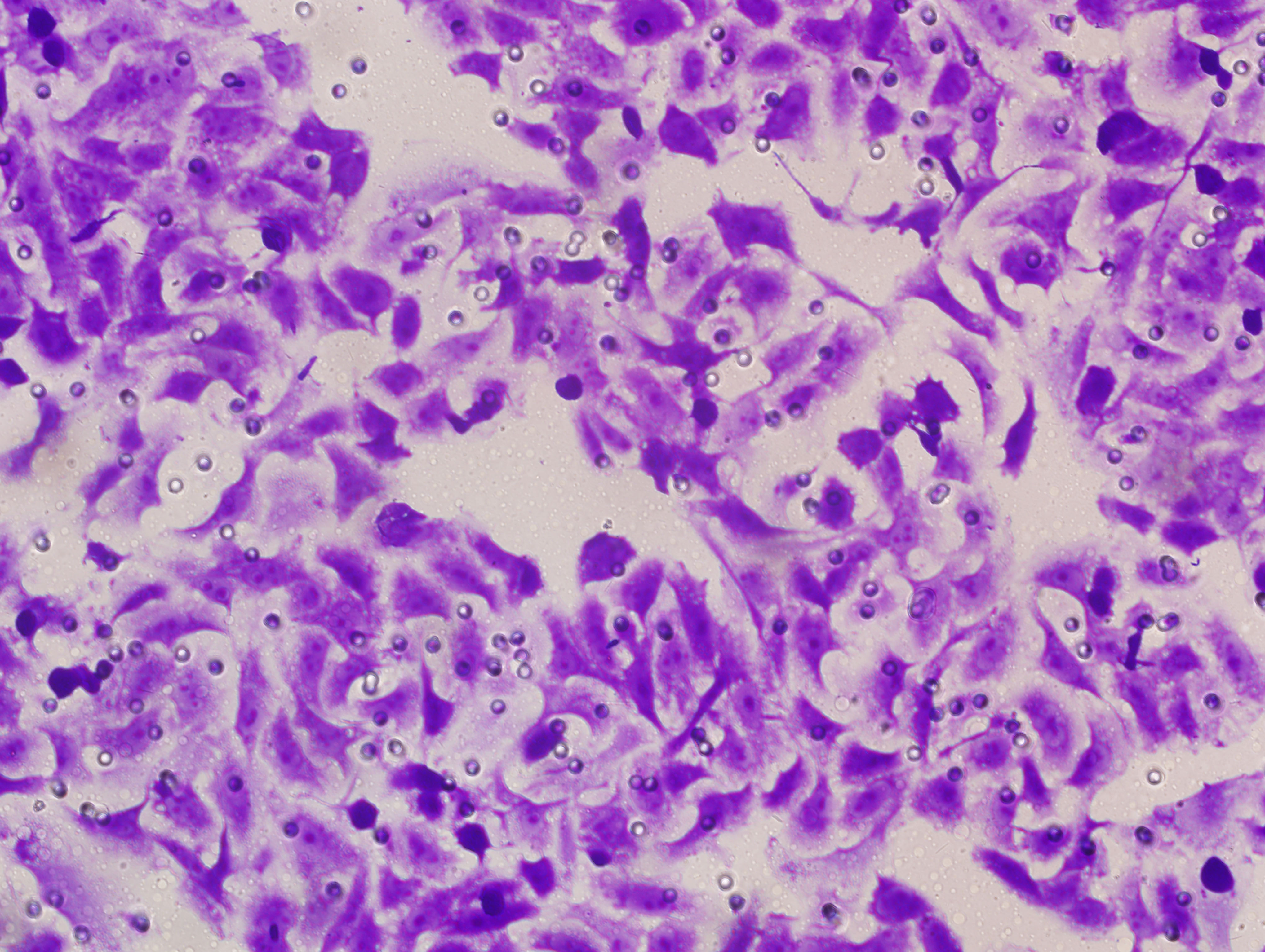

Supplement: Supplementary file 4 [file DataSheet4.zip › Invasion/Invasion SCR.jpg]

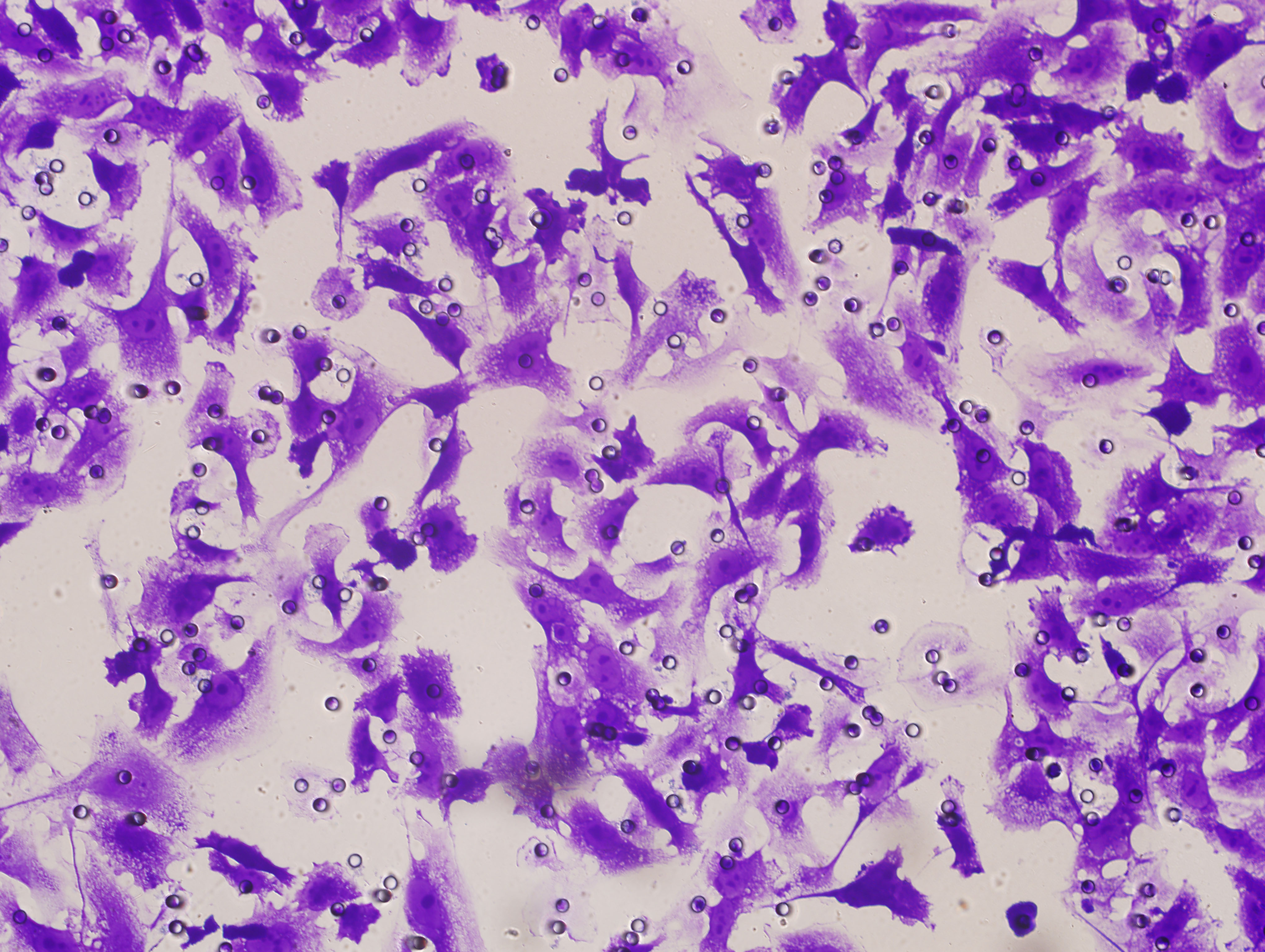

Supplement: Supplementary file 5 [file DataSheet5.zip › Migration/Migration KD.jpg]

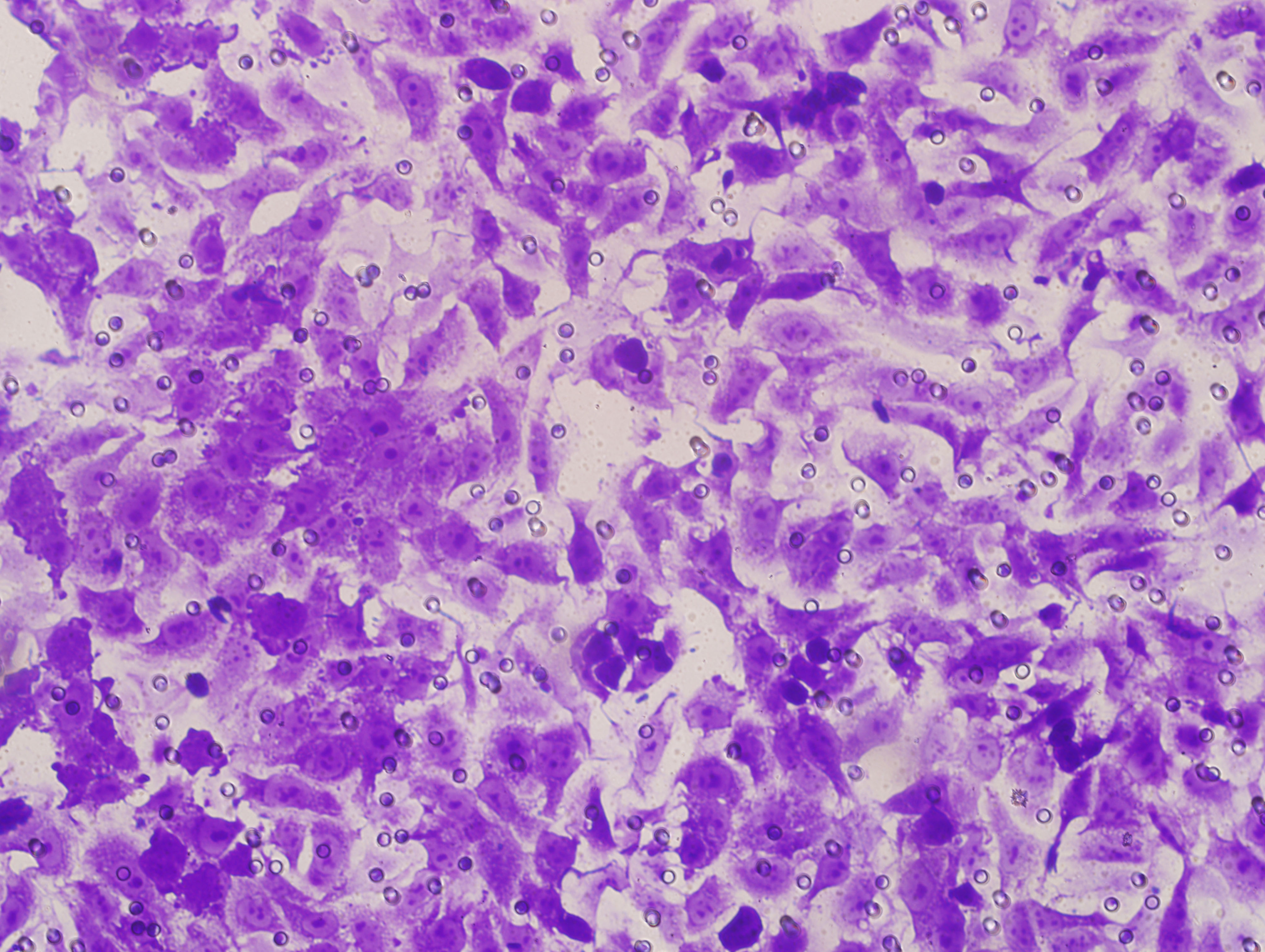

Supplement: Supplementary file 5 [file DataSheet5.zip › Migration/Migration SCR.jpg]

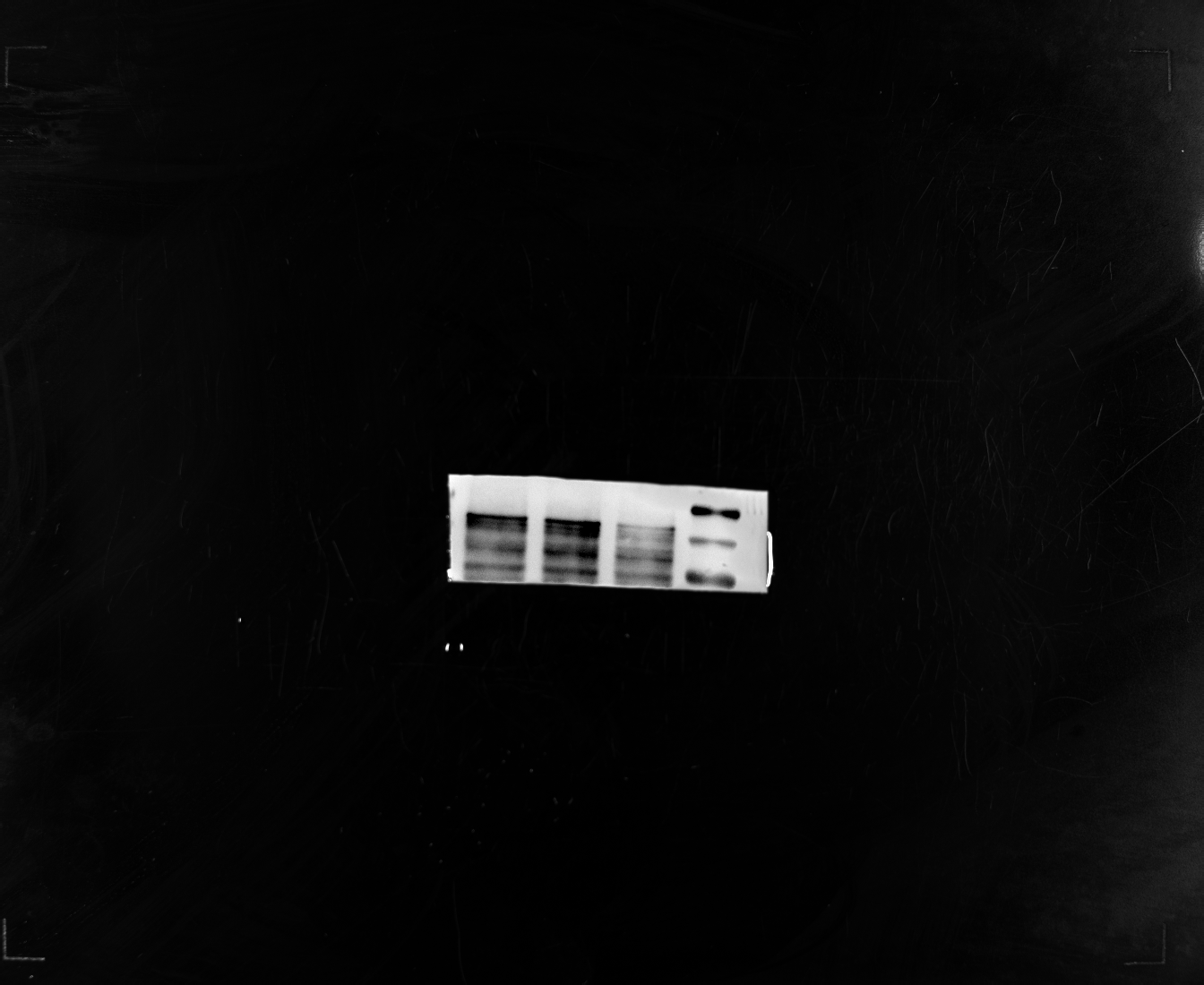

Supplement: Supplementary file 6 [file DataSheet6.zip › WB of constructed cell lines/010-merger[LARS1].tif]

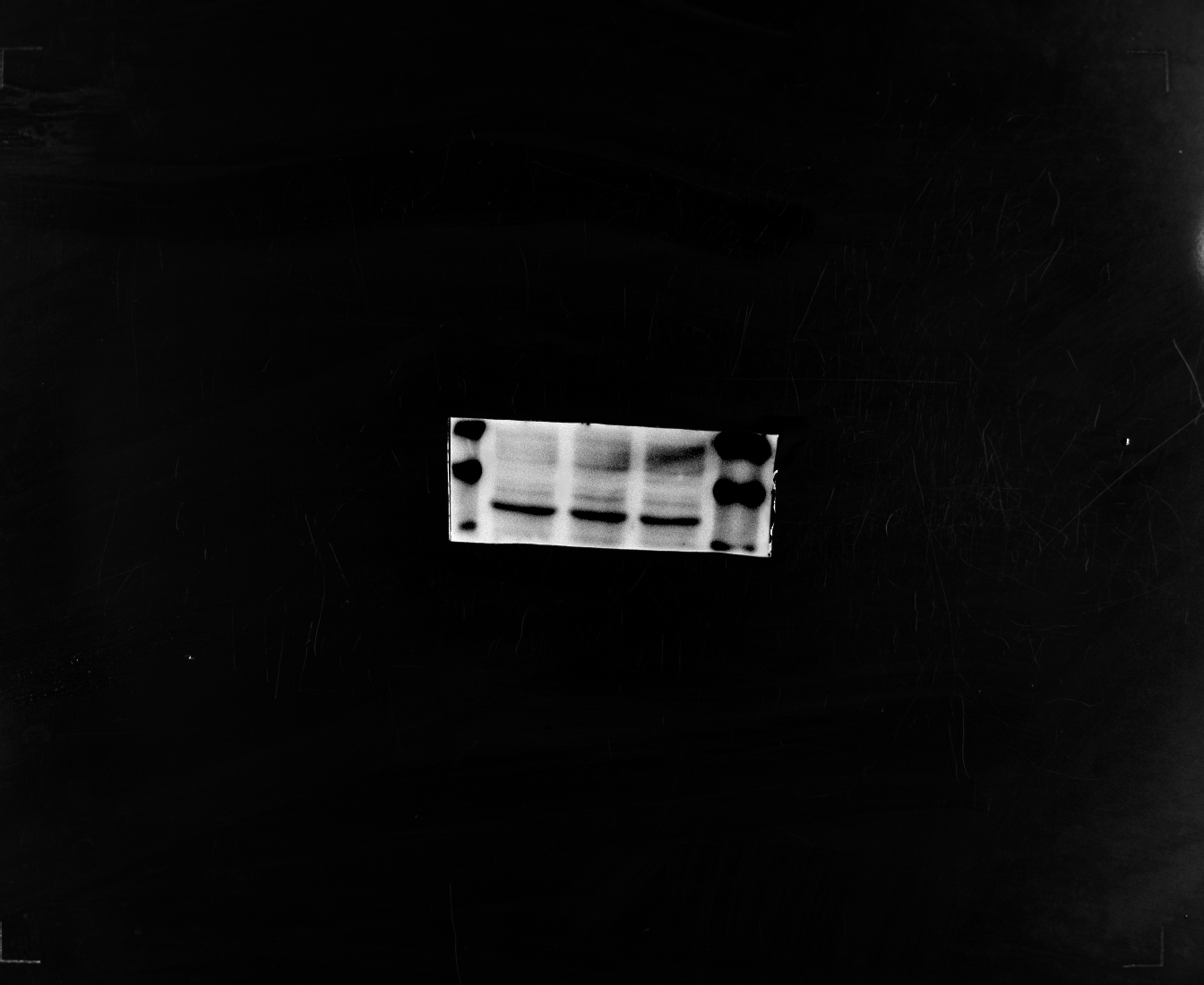

Supplement: Supplementary file 6 [file DataSheet6.zip › WB of constructed cell lines/012-merger[bactin].tif]

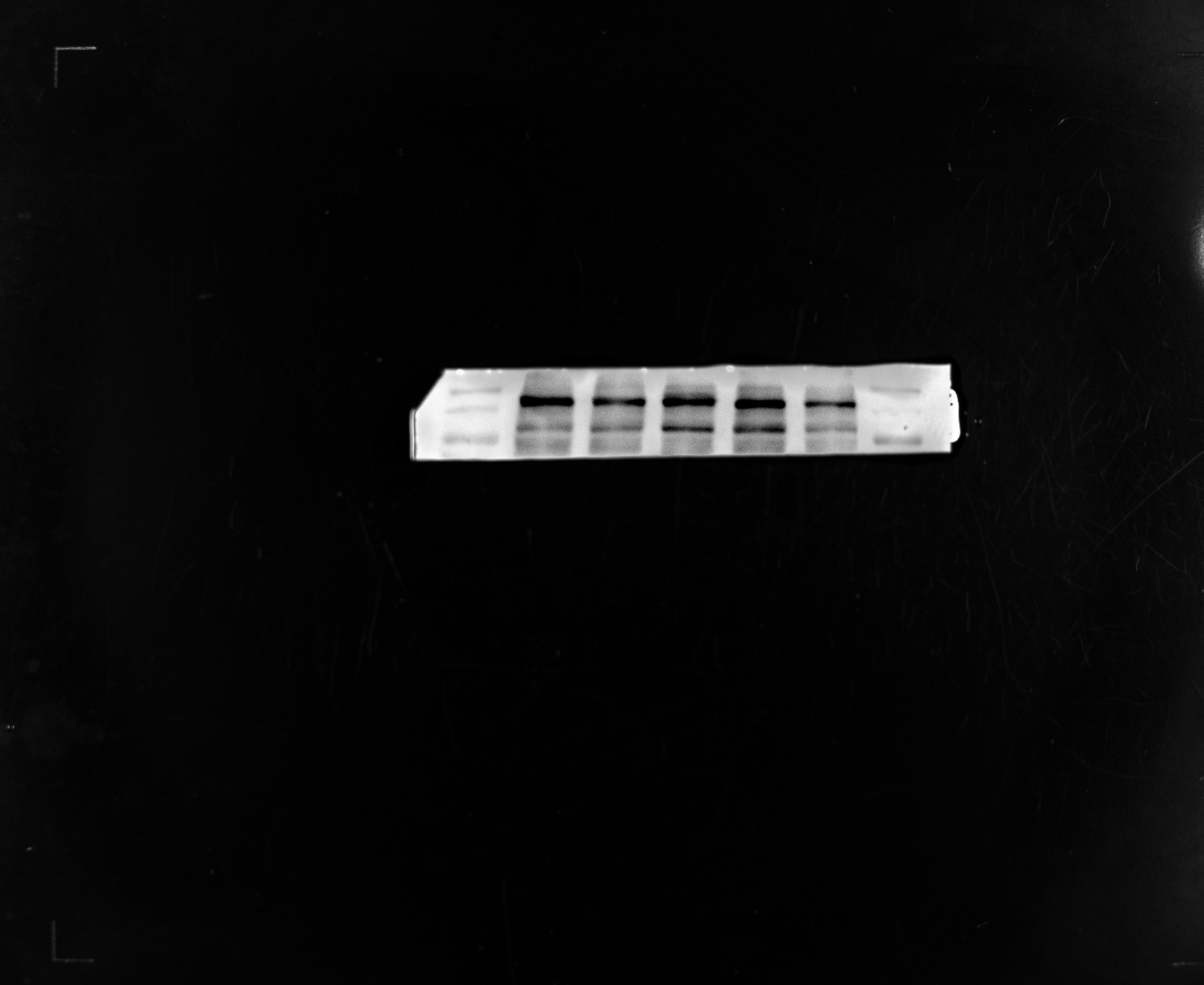

Supplement: Supplementary file 7 [file DataSheet7.zip › WB of LARS1 in different HCC cell lines/003-merger[LARS1].tif]

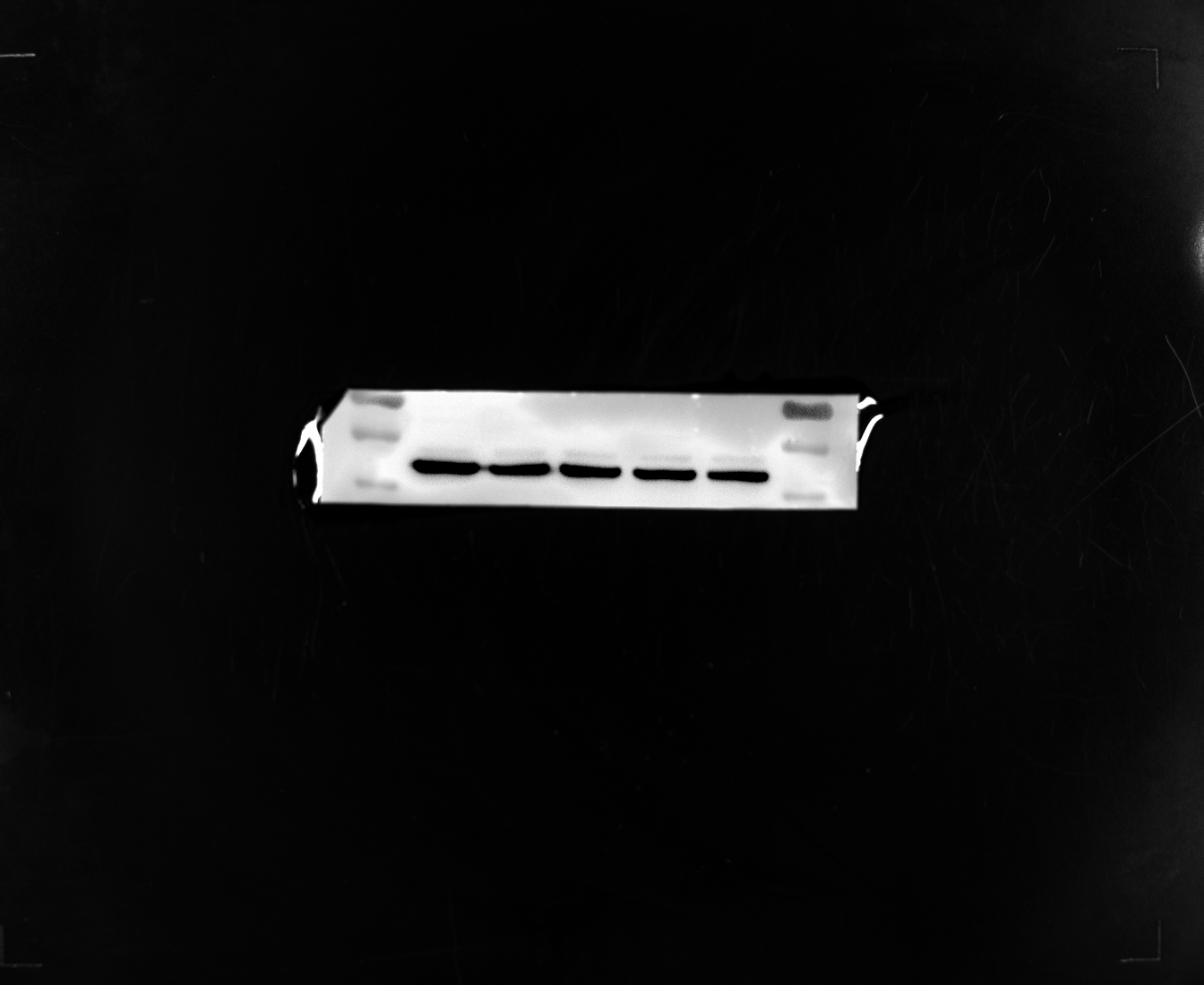

Supplement: Supplementary file 7 [file DataSheet7.zip › WB of LARS1 in different HCC cell lines/004-merger[bactin].tif]

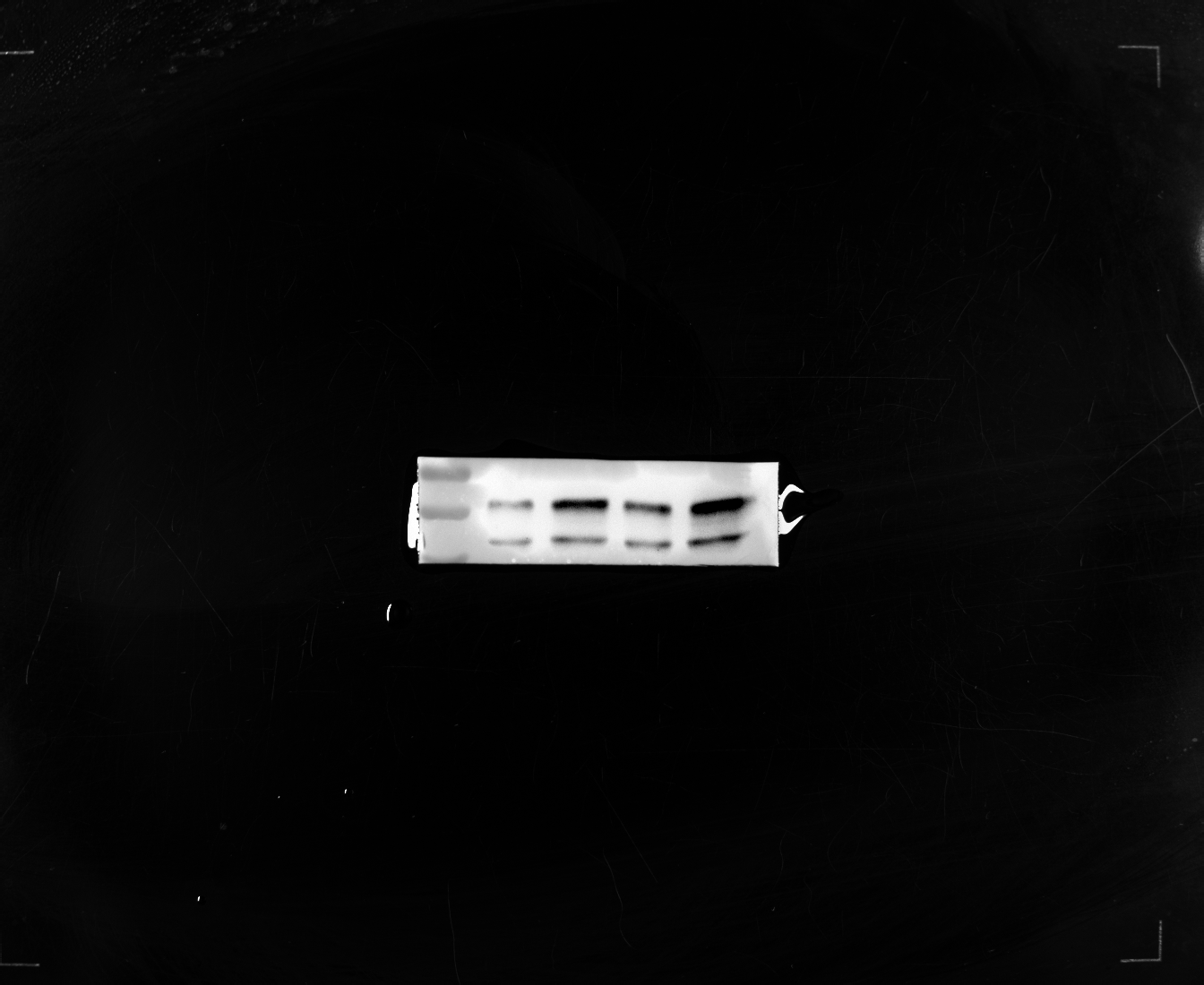

Supplement: Supplementary file 8 [file DataSheet8.zip › WB of autophagy/005-merger[ATG5].tif]

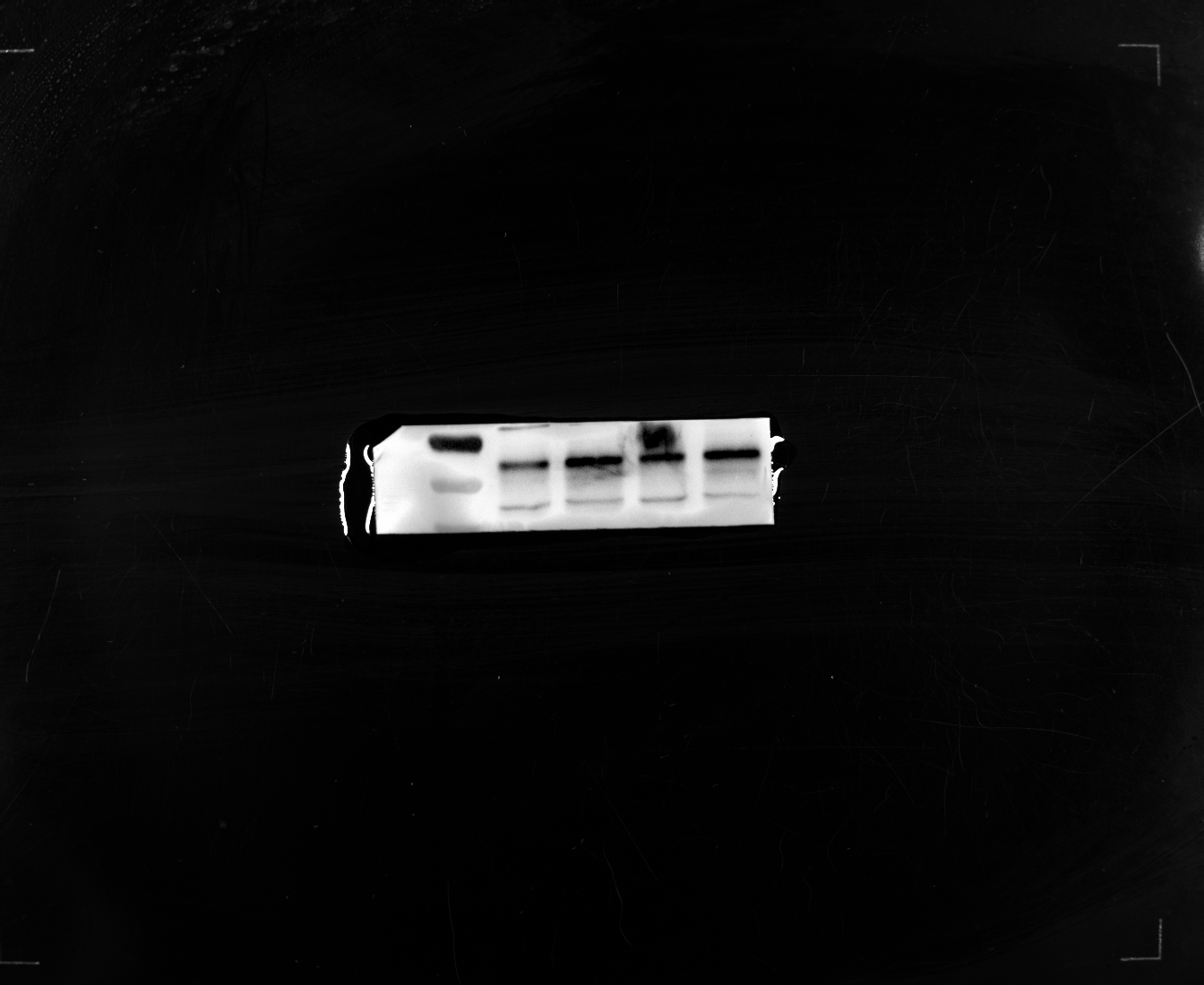

Supplement: Supplementary file 8 [file DataSheet8.zip › WB of autophagy/006-merger[Beclin1].tif]

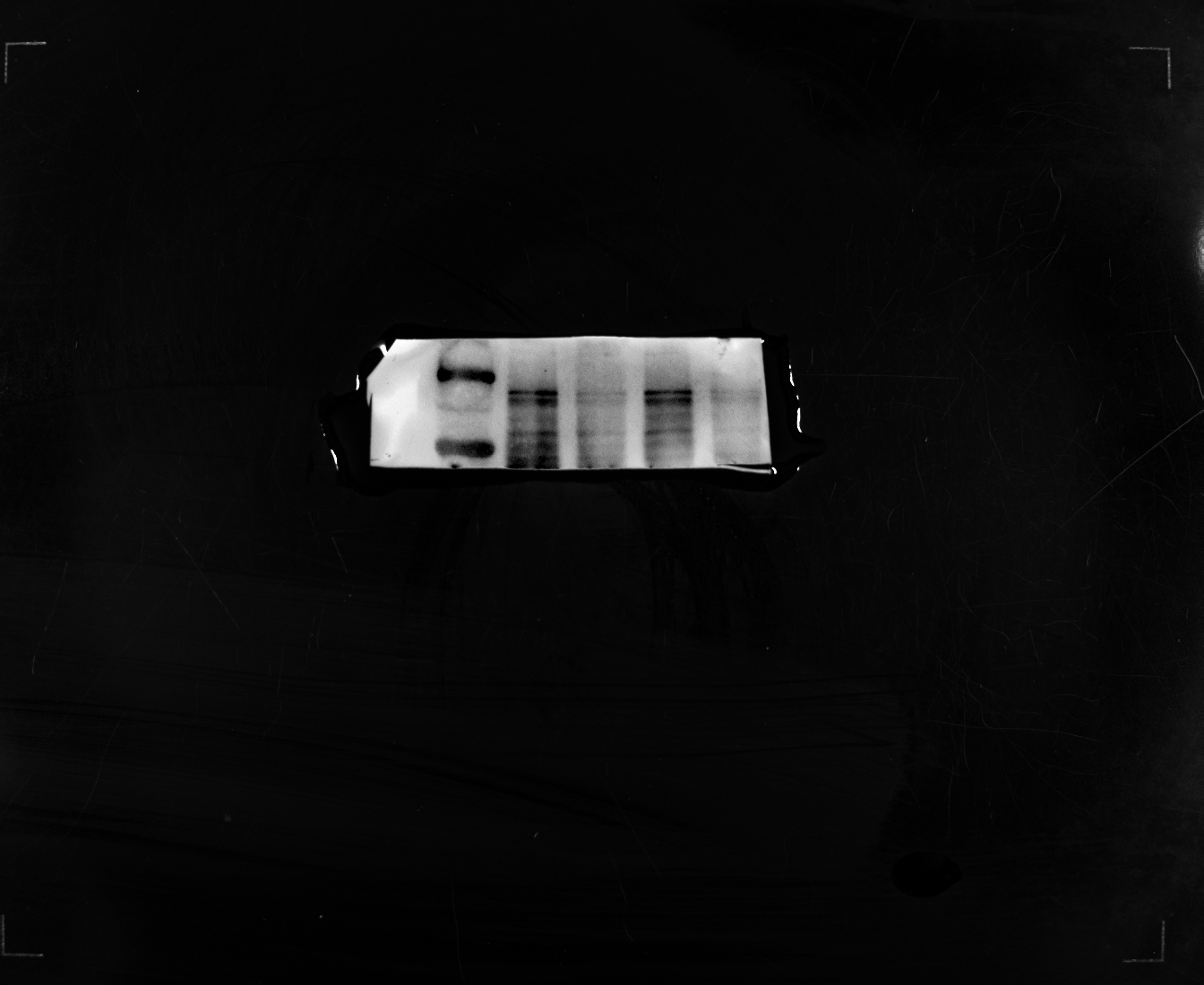

Supplement: Supplementary file 8 [file DataSheet8.zip › WB of autophagy/006-merger[LARS1].tif]

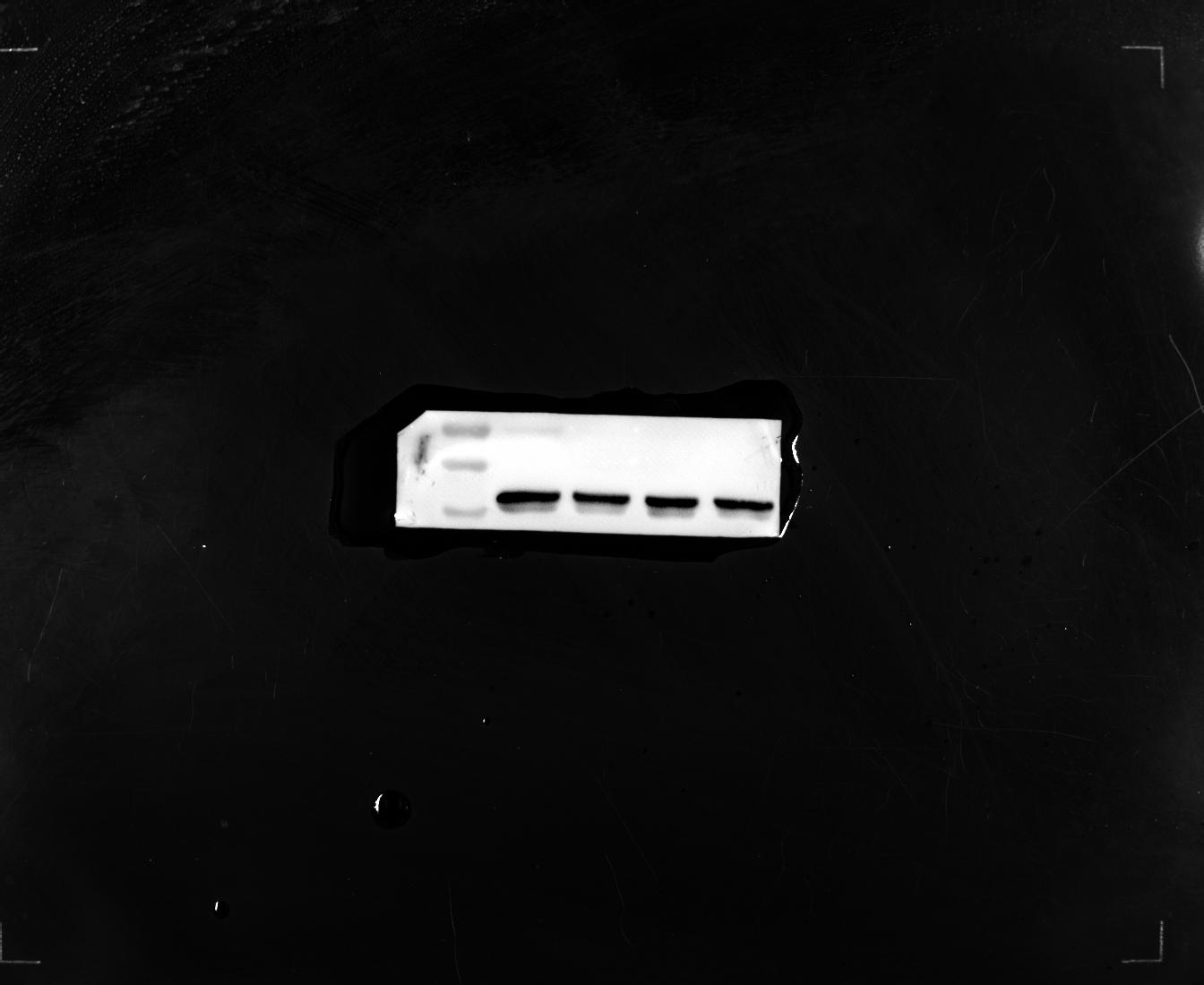

Supplement: Supplementary file 8 [file DataSheet8.zip › WB of autophagy/007-merger[bactin].tif]

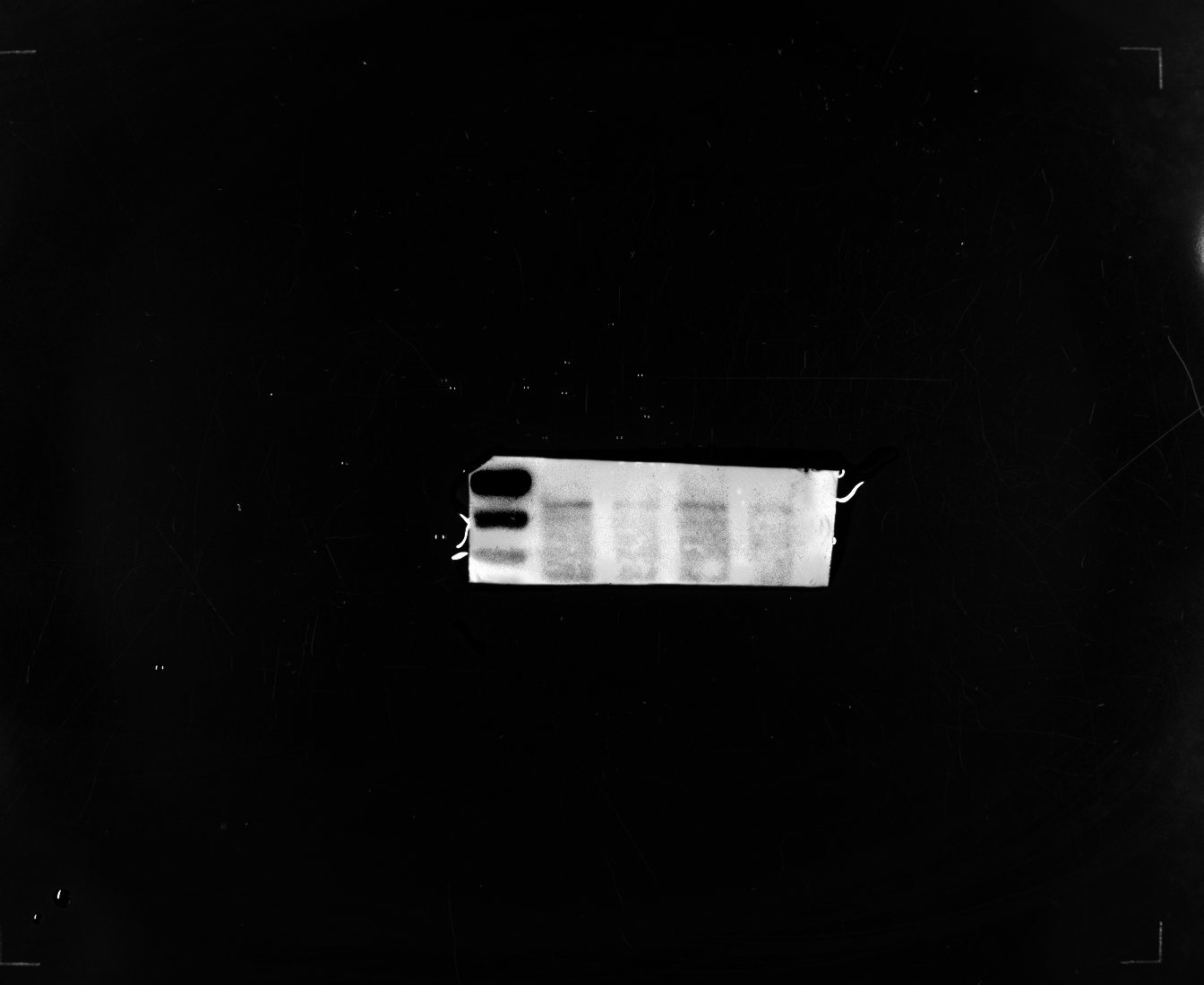

Supplement: Supplementary file 8 [file DataSheet8.zip › WB of autophagy/011-merger[P62].tif]

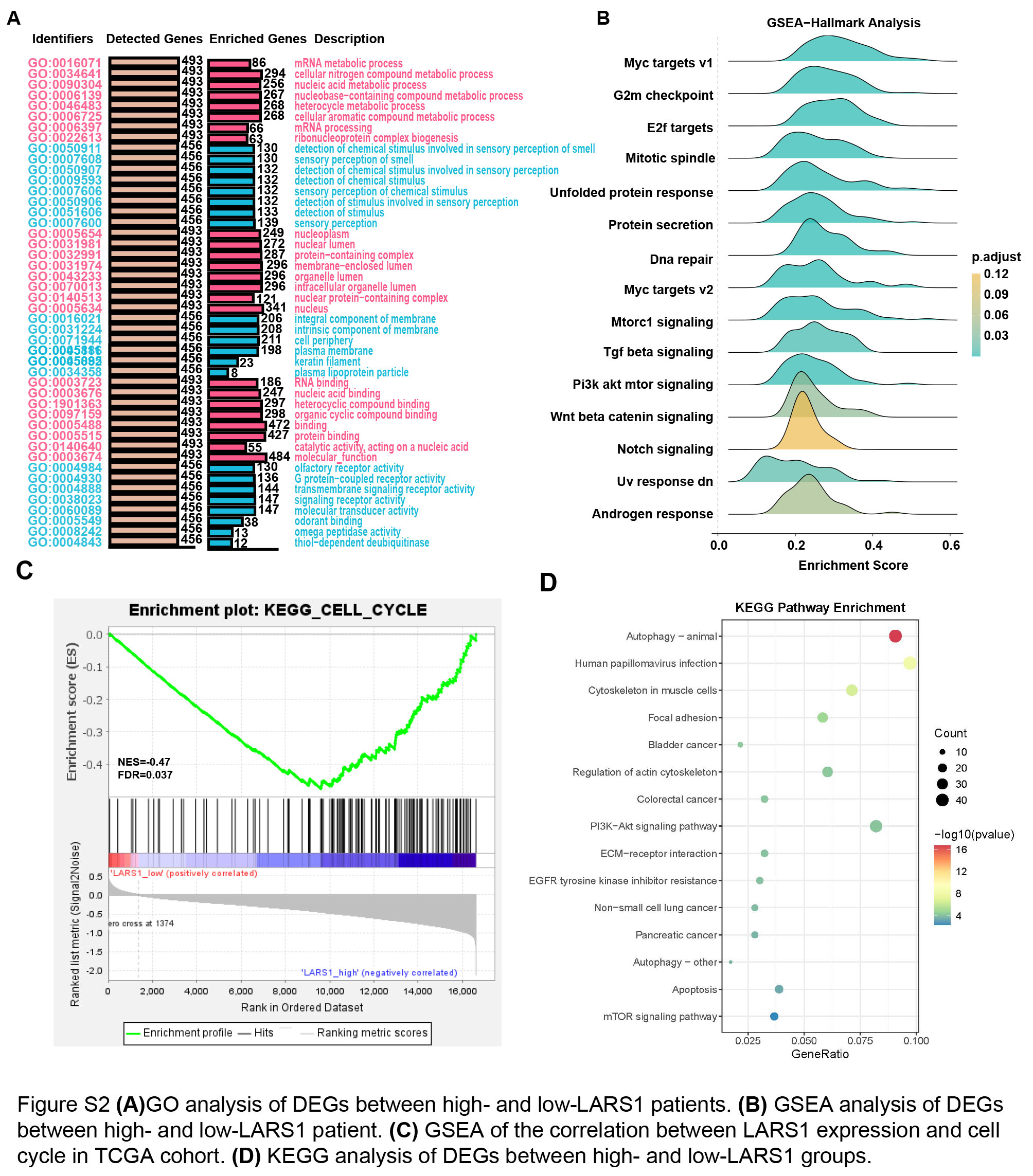

Supplement: Supplementary file 11 [file Image2.jpeg]
