# Supplementary material for: Identification of amino acid metabolism-related gene Leucyl-tRNA synthetase 1 (LARS1) as a potential prognostic and therapeutic target in hepatocellular carcinoma
Source: Front Oncol. 2025 Sep 16;15:1675018. doi: 10.3389/fonc.2025.1675018 (PMC12479282; doi:10.3389/fonc.2025.1675018)
Supplement: Supplementary file 9 [file Supplementaryfile1.docx]

Table S1 Amino acid-related genes from MSigDB database

Table S2 Selected amino acid metabolism-related genes after intersection

Data Sheet S1 Code files, data sheets from from public database and original data for CCK8 and PCR assays

Data Sheet S2 The original pictures of IHC assays displayed in manuscript

Data Sheet S3 The original pictures of EDU assays displayed in manuscript

Data Sheet S4 The original pictures of Invasion assays displayed in manuscript

Data Sheet S5 The original pictures of Invasion assays displayed in manuscript

Data Sheet S6 The original files of WB for constructing cell lines

Data Sheet S7 The original files of WB for LARS1 in different HCC cell lines

Data Sheet S8 The original files of WB for autophagy

Figure S1 CDF curve in Consensus Clustering

Figure S2 (A)Go analysis of DEGs between high- and low-LARS1 patients. (B) GSEA analysis of DEGs between high- and low-LARS1 patients. (C)GSEA analysis of DEGs between LARS1 expression and cell cycle in TCGA cohort. (D)KEGG analysis of DEGs between high- and low-LARS1 groups.
